# Pestivirus bovine viral diarrhea virus infection induces ROS–HIF-1a axis-driven glycolytic reprogramming, which increases viral replication by impairing RIG-I-dependent type I interferon response

**DOI:** 10.1128/jvi.00320-26

**Published:** 2026-05-07

**Authors:** Yuan Li, Jiangfei Zhou, Jing Wang, Kai Yan, Yueming Guan, Mengmeng Wang, Jiayi Xiang, Yimei Liu, Han Yu, Shuo Jia, Wentao Yang, Yigang Xu (徐义刚)

**Affiliations:** 1 Guangdong Engineering Technology Research Center of Biosafety and Intelligent Control for Aquatic Animals Diseases, Zhongkai University of Agriculture and Engineering, College of Animal Science & Technology 547855, Guangzhou, China; 2 Key Laboratory of Applied Technology on Green-Eco-Healthy Animal Husbandry of Zhejiang Province, Zhejiang A&F University, College of Animal Science & Technology 722545, Hangzhou, China; 3 Jilin Agricultural University, College of Veterinary Medicine 623721, Changchun, China; 4 Key Laboratory for Animal Disease Control and Pharmaceutical Development of Heilongjiang Province, Northeast Agricultural University 12430, Harbin, China; The University of Texas Southwestern Medical Center, Dallas, Texas, USA

**Keywords:** bovine viral diarrhea virus, glycolysis reprogramming, lactate, RIG-I/MAVS pathway, type I interferon

## Abstract

Pestivirus bovine viral diarrhea virus (BVDV) is a major causative agent of bovine viral diarrhea-mucosal disease, responsible for substantial economic losses in the global cattle industry. BVDV employs sophisticated strategies to evade host antiviral innate immune responses; however, the precise mechanisms remain incompletely understood. In this study, we demonstrate that BVDV infection induces HIF-1α-mediated glycolytic reprogramming, which, in turn, antagonizes the RIG-I/MAVS pathway and suppresses type I interferon (IFN-I) production, thereby facilitating viral replication. We show that BVDV infection activates endoplasmic reticulum stress, leading to a marked increase in reactive oxygen species (ROS) that promote both the expression and stabilization of HIF-1α. As a key regulator of glycolysis, nuclear translocation of HIF-1α upregulates glycolysis-related proteins, including GLUT1, PFKP, HK2, and LDHA, thereby enhancing glycolytic flux. Furthermore, BVDV-induced glycolysis stimulates the formation of an HK2/MAVS/VDAC1 complex, which disrupts RIG-I–MAVS interaction and impairs pathway activation, inhibiting IFN-I production. Additionally, we found that lactate, a glycolytic byproduct, competitively binds to MAVS, impedes its mitochondrial localization, and consequently disrupts the engagement between RIG-I and MAVS. Collectively, our findings reveal a novel mechanism by which BVDV exploits the ROS−HIF-1α−glycolysis axis to attenuate MAVS-mediated antiviral signaling and promote viral replication.

**IMPORTANCE:**

Bovine viral diarrhea virus (BVDV), a member of the genus Pestivirus, is the causative agent of bovine viral diarrhea-mucosal disease, one of the most significant infectious diseases affecting cattle worldwide. BVDV employs diverse mechanisms to evade host innate antiviral immune response, while the precise processes remain incompletely understood. Here, we reveal that BVDV infection drives glycolytic reprogramming through the ROS−HIF-1α axis, leading to the formation of an HK2/MAVS/VDAC1 complex. This complex impairs the interaction between RIG-I and MAVS, resulting in suppressed IFN production. Moreover, we show that lactate, produced via LDHA-mediated glycolysis, binds to MAVS, inhibiting its mitochondrial localization and subsequent association with RIG-I. Together, these mechanisms reveal how BVDV harnesses glycolytic remodeling to dampen RIG-I/MAVS signaling and facilitate viral replication. Our study not only uncovers a potential therapeutic target for combating pestivirus infection but also provides valuable insights into immune evasion strategies shared within the Flaviviridae family, particularly among pestiviruses.

## INTRODUCTION

Bovine viral diarrhea virus (BVDV), a member of the genus *Pestivirus* within the family *Flaviviridae*, has a positive-sense single-stranded RNA genome approximately 12.9 kb in length. It is the causative agent of bovine viral diarrhea-mucosal disease, a major global disease of cattle characterized by clinical signs such as severe enteritis, mucosal erosion and necrosis, immunosuppression, and persistent infection. BVDV infection leads to substantial economic losses in the cattle industry worldwide (1–3). Persistently infected (PI) cattle, which shed the virus throughout their lifetime, are considered the primary reservoir for maintaining and spreading BVDV within cattle populations (4–6). The virus exhibits a broad host tropism, capable of infecting various cloven-hoofed animals, including pigs, sheep, and deer (7–10). Current strategies to control and prevent BVDV infection rely on vaccination programs and the identification and removal of PI animals (11, 12). Therefore, a detailed understanding of the mechanisms by which BVDV evades host innate antiviral immunity is crucial for developing effective vaccines and therapeutic interventions against BVDV.

Type I interferon (IFN-I) plays a central role in the host’s innate antiviral immune responses by upregulating the expression of various antiviral proteins (13–15). A key mechanism triggering this response involves the recognition of viral RNA by retinoic acid-inducible gene-I (RIG-I)-like receptors (RLRs). Following viral RNA detection, RLRs activate mitochondrial antiviral signaling protein (MAVS), which, in turn, recruits tumor necrosis factor receptor-associated factor 3 (TRAF3). This complex then activates the TRAF family member-associated nuclear factor kappa B activator (TANK)-binding kinase 1 (TBK1) and IκB kinase ε (IKKε) complex. Phosphorylation of interferon regulator factors (IRFs) by TBK1 promotes the production of IFN-I and subsequent expression of IFN-stimulated genes (ISGs). These ISGs inhibit viral replication through multiple mechanisms and enhance the production of antiviral cytokine (16–18). However, many viruses have evolved sophisticated strategies to subvert RLR-mediated innate antiviral immunity, thereby facilitating viral replication and persistence (19–22).

Energy metabolism plays a fundamental role in sustaining cellular homeostasis and supporting immune responses. Upon viral infection, host cells frequently undergo extensive metabolic reprogramming to meet the heightened energy demand imposed by both antiviral defenses and viral replication processes (23–26). A hallmark of this reprogramming is aerobic glycolysis, commonly referred to as the “Warburg effect,” in which cells preferentially utilize glycolysis over oxidative phosphorylation for energy production, even under oxygen-sufficient conditions (27–29). During glycolysis, glucose is catabolized into pyruvate, which can either enter the mitochondria for oxidation via the tricarboxylic acid (TCA) cycle or be converted into lactate by lactate dehydrogenase A (LDHA), particularly under anaerobic conditions (30). HIF-1α serves as a master regulator of this metabolic shift, promoting glycolysis by upregulating key glycolytic enzymes and thereby accelerating glycolytic flux (31–33). Once considered merely as a glycolytic byproduct, lactate is now recognized as an important signaling molecule that modulates immune responses and metabolic pathways (34–36). Several viruses, including dengue virus (DENV) (37), influenza A virus (38), and hepatitis C virus (HCV) (39), have been shown to induce metabolic reprogramming that enhances lactate production, which in turn facilitates viral replication. Emerging evidence further suggests that glycolysis-derived lactate, together with glycolytic enzyme hexokinase 2 (HK2), disrupts the RIG-I–MAVS interaction, thereby suppressing IFN-I production and promoting viral replication (40–42). BVDV has also been reported to reprogram host metabolism to favor viral replication (23, 26). Our previous work demonstrated that BVDV infection upregulates key glycolytic enzymes, including HK2, pyruvate kinase (PK), and phosphofructokinase (PFK), resulting in enhanced glycolytic flux (43). However, the precise mechanisms by which BVDV exploits glycolytic reprogramming to enhance its replication remain poorly understood.

In this study, we explored the mechanisms through which BVDV infection induces glycometabolic reprogramming and modulates innate immune responses. We found that BVDV infection triggers HIF-1α-driven glycolytic reprogramming, leading to enhanced formation of a HK2/MAVS/VDAC1 (voltage-dependent anion channel 1) complex. This assembly disrupts the interaction between RIG-I and MAVS. Additionally, BVDV infection upregulates LDHA-dependent lactate production—a key glycolytic metabolite that competitively binds to MAVS and further inhibits RIG-I/MAVS signaling, thereby attenuating IFN-I production. Ore results unveil a glycolysis-mediated immune evasion strategy employed by BVDV, providing new mechanistic insights into how pestivirus BVDV manipulates host metabolism to suppress antiviral immunity.

## RESULTS

### Changes in levels of lactate and IFN-I during BVDV infection

**Fig 1:**
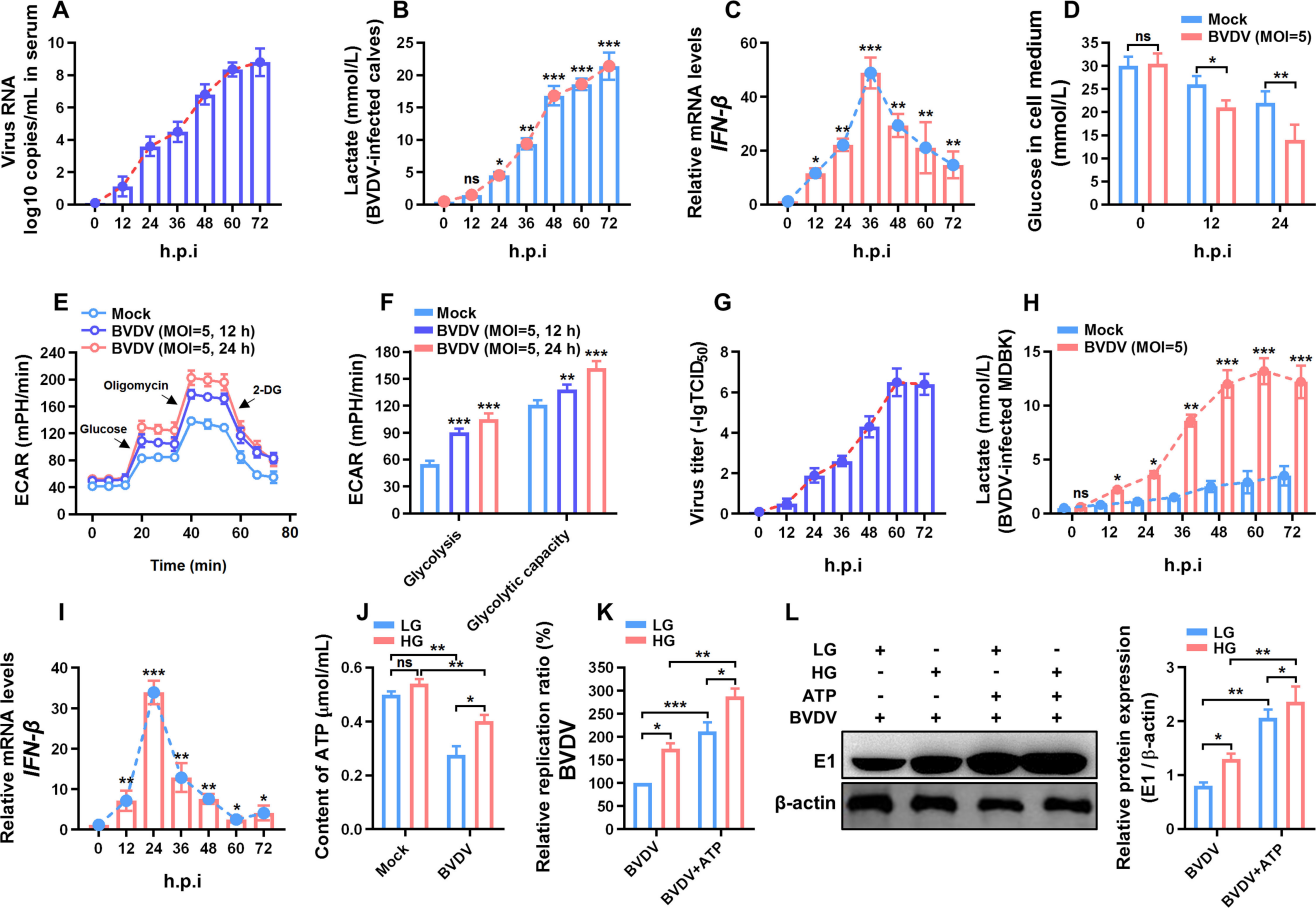
Determination of lactate production, extracellular acidification rate (ECAR), IFN-β expression level, and intracellular ATP content following BVDV infection. (**A**) Viral replication levels in infected calves. (**B**) Lactate levels in calves at various time points post-infection. (**C**) IFN-β mRNA levels in calves at different time points post-infection. (**D**) Glucose consumption in BVDV-infected MDBK cells. (**E**) ECAR was measured at 12 h and 24 h in BVDV-infected MDBK cells. (**F**) Glycolysis and glycolytic capacity in BVDV-infected MDBK cells. (**G**) Viral titer in BVDV-infected MDBK cells. (**H**) Lactate levels in BVDV-infected MDBK cells at different time points. (**I**) IFN-β mRNA levels in MDBK cells at different time points post-infection. (**J**) Intracellular ATP content in BVDV-infected MDBK cells under high-glucose (HG) and low-glucose (LG) culture conditions. (**K**) BVDV replication levels in ATP-supplemented cells under HG and LG conditions, as determined by qRT-PCR. (**L**) Viral protein expression in ATP-supplemented cells under HG and LG conditions, as assessed by western blot. Data are representative of three independent experiments and are presented as mean ± SD. *P < 0.05, **P < 0.01, ***P < 0.001; ns, not significant.

In the *in vivo* study, the healthy calves were experimentally infected with BVDV (Fig. 1A), and subsequent analyses were conducted to assess lactate production and mRNA expression levels of IFN-I in the blood of BVDV-infected calves. The findings indicated a continuous increase in the level of lactate post-BVDV infection (Fig. 1B). In contrast, the mRNA expression level of IFN-β peaked approximately 36 h following viral infection, after which it exhibited a significant decline (Fig. 1C). Further investigations involved assessing glucose consumption in BVDV-infected MDBK cells. The results demonstrated a marked reduction in glucose concentration in the culture medium of BVDV-infected MDBK cells compared to mock-infected cells (Fig. 1D). Additionally, the extracellular acidification rate (ECAR), a recognized marker of glycolysis, was evaluated. As shown in Fig. 1E, ECARs were significantly elevated upon glucose addition and further increased with oligomycin supplementation in BVDV-infected MDBK cells relative to mock-infected cells. However, ECAR levels significantly decreased following treatment with 2-DG in both mock- and BVDV-infected cells. Correspondingly, in BVDV-infected MDBK cells, both glycolysis and glycolytic capacity were significantly enhanced compared to mock-infected cells (Fig. 1F). In MDBK cells infected with BVDV, we determined the viral titer at different time points post-infection (Fig. 1G). Lactate production was markedly elevated following BVDV infection (Fig. 1H), mirroring the trend observed *in vivo*. Subsequently, we found the mRNA expression level of IFN-β in MDBK cells reached its peak at 24 h after BVDV infection and then significantly decreased (Fig. 1I). While lactate accumulation is indicative of metabolic reprogramming, the potential link between BVDV-induced metabolic reprogramming and the suppression of IFN-I expression warrants further investigation. Glucose serves as the primary energy source for cellular metabolism in animal cells, typically undergoing oxidative phosphorylation to produce ATP. Measurement of ATP dynamics in BVDV-infected cells under both high-glucose (HG) and low-glucose (LG) culture conditions revealed a significant decrease in ATP content, particularly under LG conditions (Fig. 1J). Additionally, ATP supplementation was found to markedly enhance viral replication (Fig. 1K), with a significant increase in viral protein levels (Fig. 1L). These findings indicate that BVDV replication is highly dependent on the host’s energy metabolism.

### BVDV infection promotes glycolysis via enhancing the expression and stability of HIF-1α

**Fig 2:**
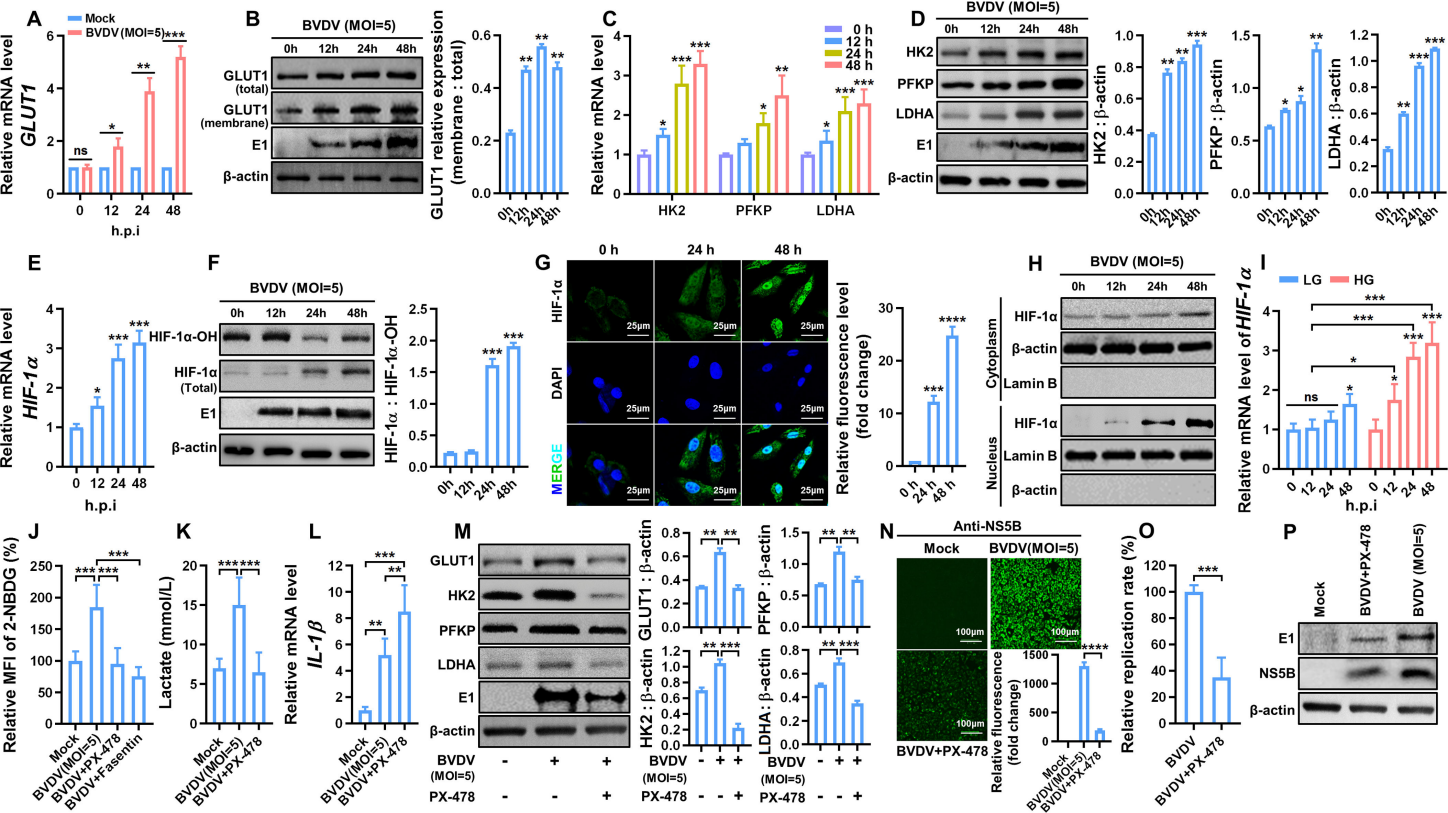
BVDV infection enhances the stability and expression of HIF-1α to promote glycolysis in infected MDBK cells. (**A**) GLUT1 mRNA levels in BVDV-infected MDBK cells. (**B**) Protein expression and cellular translocation of GLUT1 to the membrane in infected MDBK cells. (**C**) mRNA levels of HK2, PFKP, and LDHA in BVDV-infected MDBK cells at different time points post-infection. (**D**) Protein levels of HK2, PFKP, and LDHA in BVDV-infected MDBK cells following BVDV infection. (**E**) HIF-1α mRNA level in BVDV-infected cells at different time points post-infection. (**F**) Protein expression and stability of HIF-1α in BVDV-infected cells assessed by western blot. (**G**) Nuclear translocation of HIF-1α detected by IFA. (**H**) Nuclear translocation of HIF-1α analyzed by western blot. (**I**) HIF-1α mRNA levels in BVDV-infected cells under HG and LG culture conditions. (**J**) Glucose uptake in BVDV-infected cells pretreated with HIF-1α inhibitor PX-478 or GLUT-1 inhibitor Fasentin. (**K**) Lactate production in BVDV-infected MDBK cells pretreated with PX-478. (**L**) IFN-β mRNA levels in BVDV-infected MDBK cells following PX-478 pretreatment. (**M**) Protein levels of GLUT1, HK2, PFKP, and LDHA in infected MDBK cells pretreated with PX-478. (**N**) BVDV replication level in PX-478-pretreated MDBK cells determined by IFA. (**O**) BVDV replication in PX-478-pretreated MDBK cells measured by qRT-PCR. (**P**) Viral protein expression in PX-478-pretreated MDBK cells was evaluated by western blot. Data are representative of three independent experiments and are presented as mean ± SD. *P < 0.05, **P < 0.01, ***P < 0.001, and ****P < 0.0001; ns, not significant.

HIF-1α plays a pivotal role in modulating glycolysis and regulating the expression of key glycolysis-related proteins, including GLUT1, HK2, PFKP (phosphofructokinase and platelet), and LDHA. Among these, GLUT1 is critical for glucose uptake, and our study demonstrated a significant, time-dependent upregulation of mRNA expression in MDBK cells infected with BVDV (Fig. 2A). For effective glucose transport, GLUT1 must translocate from the cytoplasm to the cell membrane. We investigated this translocation process and confirmed an increased presence of GLUT1 at the cell membrane in BVDV-infected MDBK cells (Fig. 2B), suggesting that BVDV infection promotes GLUT1 membrane translocation. We also assessed the expression levels of HK2, PFKP, and LDHA in BVDV-infected cells, observing a progressive, time-dependent upregulation at both mRNA (Fig. 2C) and protein (Fig. 2D) levels post-BVDV infection. Given that HIF-1α serves as a pivotal regulator of the expression of GLUT1, HK2, PFKP, and LDHA, we investigated its expression and stability in MDBK cells infected with BVDV. Our findings reveal that BVDV infection significantly increased HIF-1α expression at both the mRNA (Fig. 2E) and protein (Fig. 2F) levels in a time-dependent manner, while the level of hydroxylated HIF-1α (HIF-1α-OH) was notably reduced (Fig. 2F), suggesting that BVDV promotes the expression and stabilization of HIF-1α. We employed confocal microscopy (Fig. 2G) and western blot analysis (Fig. 2H) to assess the translocation of HIF-1α from the cytoplasm to the nucleus, and the results indicated that BVDV infection significantly promoted the nuclear translocation of HIF-1α. Additionally, we confirmed that HIF-1α expression was markedly elevated in BVDV-infected MDBK cells cultured under HG conditions compared to LG conditions (Fig. 2I).

Subsequently, MDBK cells were pretreated with PX-478 (a HIF-1α inhibitor) and Fasentin (a GLUT-1 inhibitor) prior to infection with BVDV. As illustrated in Fig. 2J, the inhibition of HIF-1α and GLUT-1 led to a significant reduction in glucose uptake during BVDV infection. Furthermore, the inhibition of HIF-1α resulted in a marked decrease in lactate production in BVDV-infected MDBK cells (Fig. 2K), while the mRNA expression level of IFN-β was significantly upregulated (Fig. 2L). We also confirmed that the inhibition of HIF-1α with PX-478 substantially reduced the expression levels of glycolysis-related proteins GLUT1, HK2, PFKP, and LDHA (Fig. 2M), suggesting that HIF-1α regulates the expression of glycolysis-related proteins. Moreover, following the HIF-1α inhibition with PX-478, BVDV replication was significantly suppressed in PX-478-treated cells, as determined by IFA (Fig. 2N), qRT-PCR (Fig. 2O), and western blot analysis (Fig. 2P).

### BVDV infection promotes the expression and stability of HIF-1α via the ER stress-ROS axis

**Fig 3:**
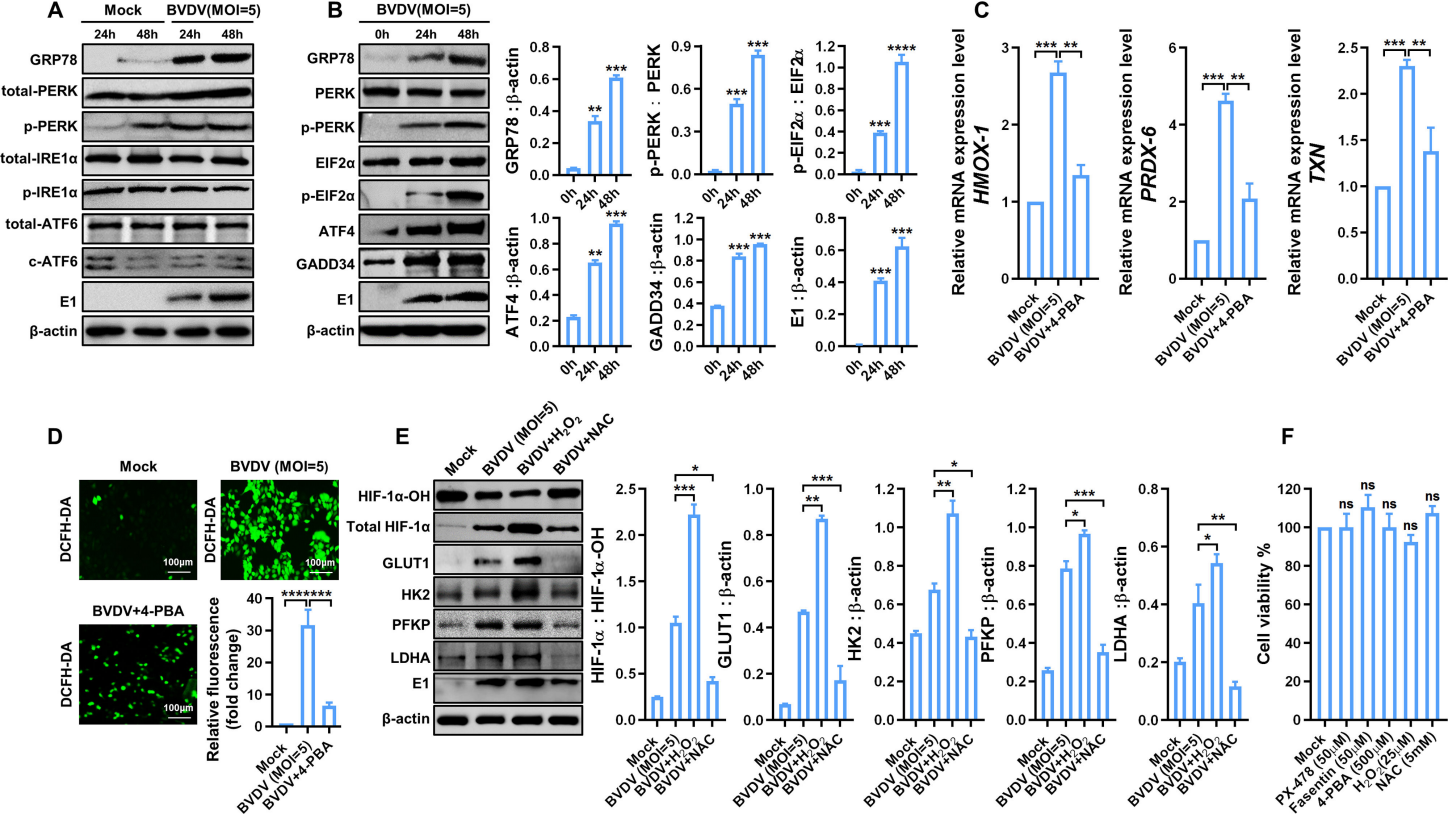
BVDV infection promotes the expression and stability of HIF-1α via the ER stress-ROS axis. (**A**) Identification of the key signaling pathway involved in BVDV-induced ER stress. (**B**) Expression of key proteins in the PERK pathway of the ER activated by BVDV infection. (**C**) Transcript levels of oxidative stress-related genes (*HMOX-1*, *TXN*, and *PRDX-6*) in BVDV-infected MDBK cells pretreated with the ER stress inhibitor 4-PBA. (**D**) ROS levels in BVDV-infected MDBK cells pretreated with 4-PBA. (**E**) Western blot analysis of HIF-1α-OH, GLUT1, HK2, PFKP, and LDHA protein levels in BVDV-infected MDBK cells pretreated with H_2_O_2_ (a ROS inducer) or N-acetylcysteine (NAC, a ROS inhibitor). (**F**) Cell viability was assessed by CCK-8 assay after treatment with various inhibitors or activators. Data are representative of three independent experiments and are presented as mean ± SD. *P < 0.05, **P < 0.01, ***P < 0.001, and ****P < 0.0001; ns, not significant.

The unfolded protein response (UPR) comprises a conserved set of intracellular signaling pathways primarily aimed at restoring endoplasmic reticulum (ER) homeostasis in response to ER stress. We analyzed the protein expression levels of GRP78 (glucose-regulated protein, 78 kDa), an ER-resident chaperone and key regulator of UPR, at 24 h and 48 h post-BVDV infection. The result revealed a substantial increase in GRP78 expression in BVDV-infected MDBK cells compared to the mock-infected control group (Fig. 3A). We further examined the signaling pathways activating ER stress and observed a marked upregulation in the phosphorylation of double-stranded RNA-dependent protein kinase R (PKR)-like ER kinase (PERK) (p-PERK). In contrast, levels of phosphorylated inositol-requiring enzyme 1α (IRE1α) and cleaved activating transcription factor 6 (ATF6) showed no significant changes (Fig. 3A). These findings indicate that BVDV infection primarily activates ER stress through the PERK signaling pathway. Subsequently, we observed that phosphorylation of eukaryotic translation initiation factor 2α (EIF2α), a downstream molecule of PERK, and expression of ATF4 and growth arrest and DNA damage-inducible protein (GADD34), key PERK effectors, were all markedly upregulated upon BVDV infection (Fig. 3B). Additionally, to investigate the role of ER stress, we treated MDBK cells with the ER stress inhibitor 4-phenylbutyrate (4-PBA). We then analyzed the expression levels of oxidative stress-related genes *HMOX-1* (heme oxygenase 1), *TXN* (thioredoxin), and *PRDX-6* (peroxiredoxin-6) in BVDV-infected MDBK cells. We found that inhibition of ER stress remarkably suppressed the expression of the genes *HMOX-1*, *TXN*, and *PRDX-6* (Fig. 3C). Previous research has indicated that reactive oxygen species (ROS), predominantly originating from mitochondria, play an important role in regulating the stability of HIF-1α (44). In this study, we assessed ROS production in BVDV-infected MDBK cells and found a significant increase in ROS level post-infection, while treatment with 4-phenylbutyric acid (4-PBA, an ER stress inhibitor) effectively inhibited BVDV-induced ROS production (Fig. 3D). Subsequently, we found that treatment with hydrogen peroxide (H_2_O_2_), a ROS inducer, enhanced the expression and activation of HIF-1α by reducing the levels of hydroxylated HIF-1α (HIF-1α-OH). This led to increased expression of GLUT1, HK2, PFKP, and LDHA (Fig. 3E). In contrast, treatment with acetylcysteine (NAC, a ROS inhibitor) suppressed HIF-1α expression and activation by elevating HIF-1α-OH levels, thereby downregulating the expression of GLUT1, HK2, PFKP, and LDHA (Fig. 3E). In conclusion, these results indicate that BVDV infection promotes HIF-1α stability and activity by inducing ROS production. The effects of inhibitors and activators on cell activity were assessed and are presented in Fig. 3F.

### BVDV inhibits the RLR signaling pathway by glycolysis

**Fig 4:**
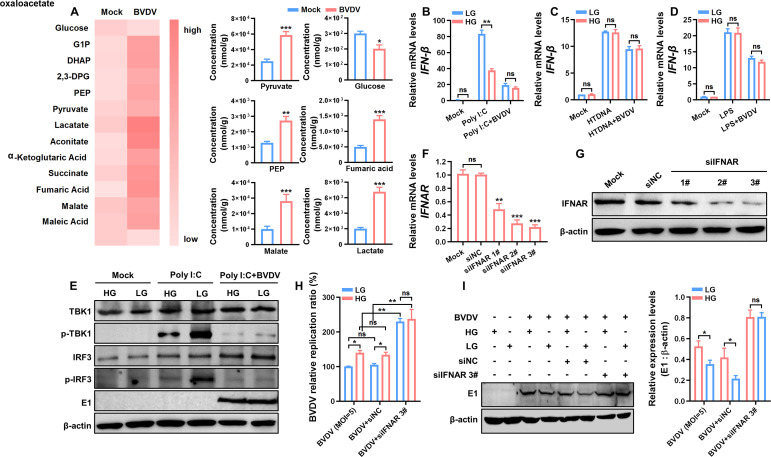
BVDV inhibits the RLR signaling pathway through glycolysis. (**A**) Levels of glucose metabolites in MDBK cells at 48 h post-BVDV infection. (**B**) IFN-β mRNA expression in poly(I:C)-transfected MDBK cells cultured under HG or LG conditions at 48 h post-infection. (**C**) IFN-β mRNA expression in HTDNA-transfected MDBK cells under HG or LG conditions at 48 h post-infection. (**D**) IFN-β mRNA expression in LPS-treated MDBK cells under HG or LG conditions at 48 h post-infection. (**E**) Protein levels of TBK1 and phospho-TBK1 (p-TBK1), IRF3, and p-IRF3 in the RLR pathway in poly(I:C)-transfected MDBK cells under HG or LG conditions at 48 h post-infection. (**F**) Knockdown efficiency of siRNAs targeting *IFNAR* as measured by qRT-PCR. (**G**) Knockdown efficiency of siRNAs targeting *IFNAR* as determined by western blot. (**H**) BVDV replication levels measured by qRT-PCR, and (**I**) viral protein expression was detected by western blot in IFNAR-knockdown MDBK cells under HG or LG conditions at 48 h post-infection. Data are representative of three independent experiments and are presented as mean ± SD. *P < 0.05, **P < 0.01, ***P < 0.001; ns, not significant.

The concentrations of glucose metabolites were quantified to assess the status of glucose metabolism in BVDV-infected MDBK cells. Our findings indicated that a significant increase in the levels of metabolites such as pyruvate, G1P, DHAP, 2,3-DPG, PEP, succinate, fumaric acid, α-ketoglutaric acid, malate, and maleic acid following BVDV infection, with a particularly notable elevation in lactate levels. Conversely, the level of oxaloacetate was significantly reduced, suggesting that BVDV infection enhances glycolytic activity (Fig. 4A). Subsequently, we investigated the effect of BVDV-induced glycolysis on the host’s innate immune response. MDBK cells cultured under HG and LG conditions were treated with HT-DNA (an agonist of the cGAS-STING pathway), LPS (an agonist of the TLR pathway), and poly(I:C) (an agonist of the RLR pathway), followed by BVDV infection. Our results showed that the mRNA expression level of IFN-β was significantly elevated in the LG + poly(I:C) group compared to the HG + poly(I:C) group (Fig. 4B), while no significant difference was observed in the mRNA expression of IFN-β between the HG group and the LG group treated with HT-DNA (Fig. 4C) or LPS (Fig. 4D). These findings suggest that glycolysis suppresses type I interferon production mediated by the RLR pathway. Additionally, the primary proteins associated with the RLR pathway were analyzed, revealing that, in comparison to the LG group, the phosphorylation levels of TANK-binding kinase 1 (TBK1) and interferon regulatory factor 3 (IRF3) were significantly reduced in the HG group treated with poly(I:C) (Fig. 4E). This indicates that glycolysis inhibits the activation of the RLR signaling pathway. To further investigate the effect of glycolysis-regulated IFN-I production on BVDV replication, siRNAs targeting the IFN-I receptor (IFNAR) were designed, and the knockdown efficiency was evaluated using qRT-PCR (Fig. 4F) and western blot analysis (Fig. 4G). Subsequently, the replication capacity of BVDV was assessed in IFNAR-knockdown MDBK cells cultured under HG and LG conditions. The findings indicated a significant reduction in BVDV replication in cells maintained under LG conditions compared to those under HG conditions. However, following IFNAR knockdown, there was a marked increase in both BVDV replication (Fig. 4H) and viral protein expression (Fig. 4I), with no significant difference observed between the LG and HG groups.

### BVDV infection promotes HK2-MAVS-VDAC1 complex formation to inhibit RLR-MAVS pathway activation

**Fig 5:**
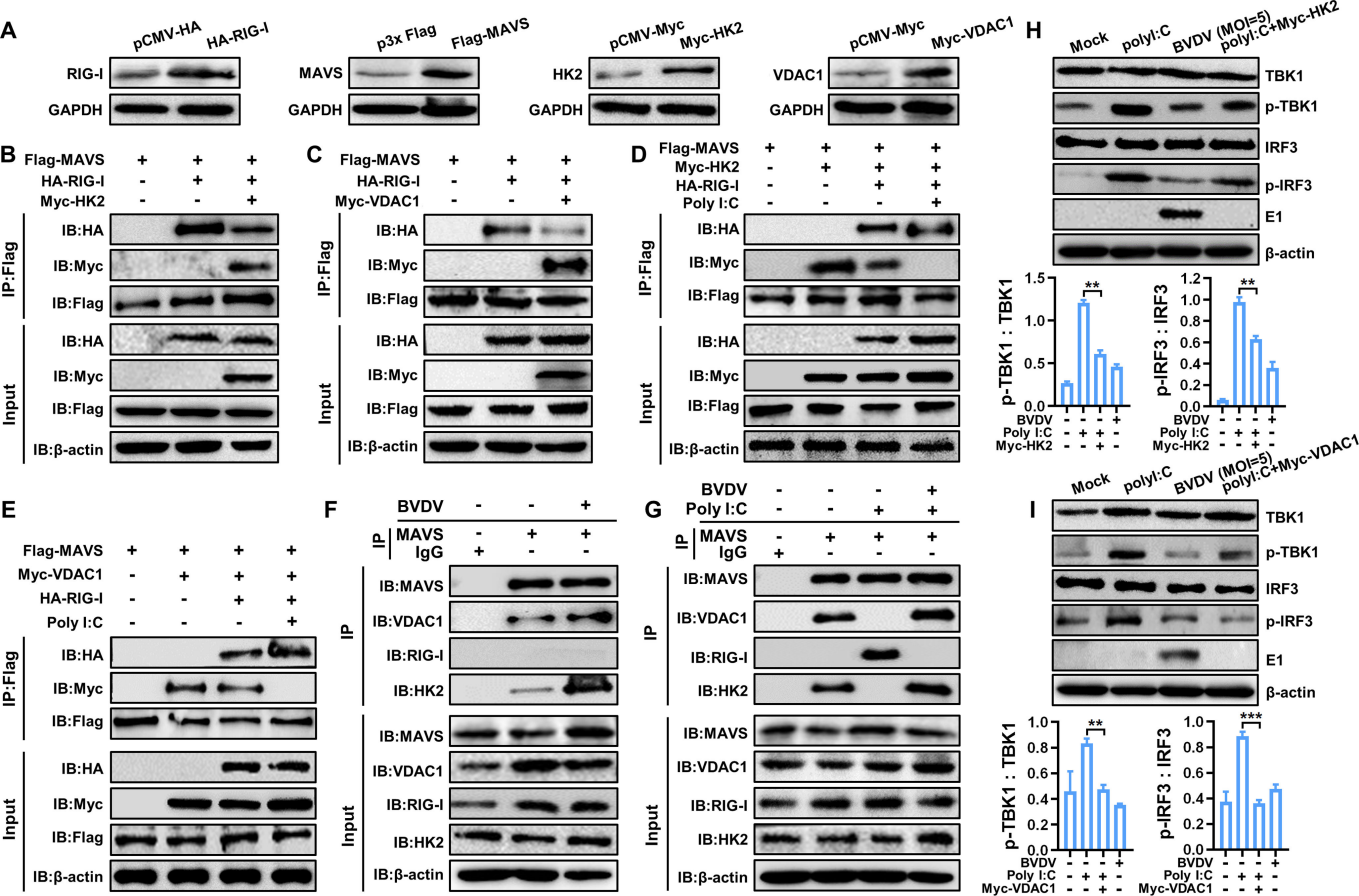
BVDV infection promotes the formation of the HK2-MAVS-VDAC1 complex to inhibit RLR-MAVS pathway activation. (**A**) Validation of plasmid expression for RIG-I, MAVS, HK2, and VDAC1 by western blot using protein-specific antibodies. (**B**) Co-IP analysis showing HK2-mediated inhibition of RIG-I/MAVS interaction in BT cells co-transfected with Myc-HK2, HA-RIG-I, and Flag-MAVS. (**C**) Co-IP analysis of VDAC1-mediated inhibition of RIG-I/MAVS interaction in BT cells co-transfected with Myc-VDAC1, HA-RIG-I and Flag-MAVS. (**D**) Disruption of the HK2/MAVS interaction upon poly(I:C) treatment, as assessed by Co-IP. (**E**) Disruption of the VDAC1/MAVS interaction upon poly(I:C) treatment, as assessed by Co-IP. (**F**) Suppression of endogenous RIG-I/MAVS interaction by the BVDV-induced HK2-MAVS-VDAC1 complex, analyzed by Co-IP. (**G**) Disruption of poly(I:C)-mediated endogenous RIG-I/MAVS interaction by the BVDV-induced HK2-MAVS-VDAC1 complex, analyzed by Co-IP. (**H**) Protein levels of TBK1, p-TBK1, IRF3, and p-IRF3 in HK2-overexpressing BT cells following poly(I:C) transfection. (**I**) Protein levels of TBK1, p-TBK1, IRF3, and p-IRF3 in VDAC1-overexpressing MDBK cells following poly(I:C) transfection. Data are representative of three independent experiments and are presented as mean ± SD. *P < 0.05, **P < 0.01, ***P < 0.001; ns, not significant.

**Fig 6:**
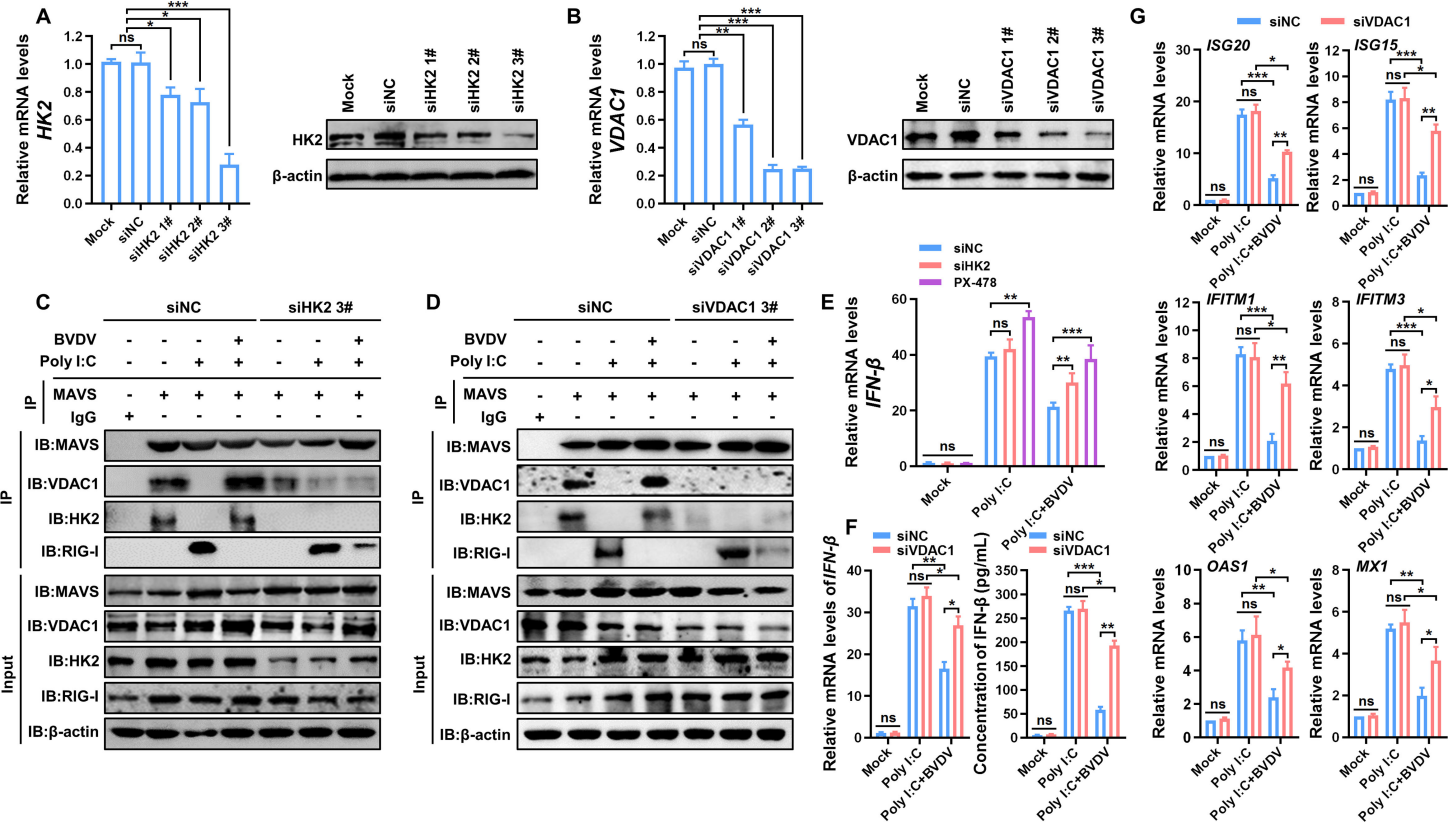
Inhibition of the HK2-MAVS-VDAC1 complex formation enhances RLR signaling pathway-mediated IFN-β production. (**A**) Knockdown efficiency of HK2*-*targeting siRNAs as determined by qRT-PCR and western blot. (**B**) Knockdown efficiency of VDAC1-targeting siRNAs as determined by qRT-PCR and western blot. (**C**) Enhanced endogenous RIG-I/MAVS interaction in HK2-knockdown MDBK cells analyzed by CO-IP. (**D**) Enhanced endogenous RIG-I/MAVS interaction in VDAC1-knockdown MDBK cells analyzed by CO-IP. (**E**) Upregulation of IFN-β mRNA expression in HK2-knockdown MDBK cells measured by qRT-PCR. (**F**) Upregulation of IFN-β mRNA expression and protein secretion in VDAC1-knockdown MDBK cells measured by qRT-PCR. (**G**) With siRNA-mediated knockdown of VDAC1, the expression levels of interferon-stimulated genes (*ISG20*, *ISG15*, *IFITM1*, *IFITM3*, *OAS1*, and *MX1*) in poly I:C-pretreated MDBK cells upon BVDV infection, as measured by qRT-PCR. Data are representative of three independent experiments and are presented as mean ± SD. *P < 0.05, **P < 0.01, ***P < 0.001; ns, not significant.

To investigate the mechanisms through which BVDV enhances glycolysis to suppress the RLR signaling pathway, the recombinant eukaryotic plasmids expressing RIG-I, MAVS, VDAC1, and HK2 proteins were constructed and subsequently verified in transfected BT cells via western blot analysis with protein-specific antibody (Fig. 5A). Co-IP assay was then employed to examine protein-protein interactions. The results indicated that both HK2 (Fig. 5B) and VDAC1 (Fig. 5C) interact with MAVS, forming the HK2-MAVS-VDAC1 complex, which obstructs the interaction between RIG-I and MAVS. However, treatment with poly(I:C) effectively disrupted the interaction between HK2 and MAVS (Fig. 5D) as well as VDAC1 and MAVS (Fig. 5E). Additionally, BVDV infection was found to significantly enhance the interactions between endogenous HK2-MAVS and VDAC1-MAVS (Fig. 5F), thereby markedly inhibiting poly(I:C)-mediated interaction between endogenous RIG-I and MAVS (Fig. 5G). Furthermore, we observed that overexpression of HK2 (Fig. 5H) and VDAC1 (Fig. 5I) markedly reduced the phosphorylation levels of TBK1 and IRF3. These data elucidate that BVDV infection suppresses the activation of the RLR signaling pathway by promoting the formation of the HK2-MAVS-VDAC1 complex. To further investigate the role of BVDV-induced HK2-MAVS-VDAC1 complex formation in the suppression of host innate antiviral immune responses, siRNAs targeting *HK2* (siHK2) and *VDAC1* (siVDAC1) were designed, and the silencing efficiency of siHK2 (Fig. 6A) and siVDAC1 (Fig. 6B) was assessed using qRT-PCR and Western blot. Our results showed that siRNA-mediated knockdown of HK2 and VDAC1 effectively disrupted the interactions of endogenous MAVS-HK2 (Fig. 6C) and endogenous MAVS-VDAC1 (Fig. 6D), respectively, thereby leading to a marked enhancement of the RIG-I–MAVS interaction. Consistent with this, knockdown of HK2 or VDAC1 significantly upregulated IFN-β mRNA expression in BVDV-infected MDBK cells (Fig. 6E and F), which corresponded with increased IFN-β protein secretion as measured by enzyme-linked immunosorbent assay (ELISA) (Fig. 6F). Moreover, treatment with the HIF-1α inhibitor PX-478 also substantially elevated IFN-β transcript levels (Fig. 6E). To further confirm the role of VDAC1 in this regulatory axis, we showed that VDAC1 knockdown potentiated interferon-stimulated genes (e.g., *ISG20*, *ISG15*, *IFITM1*, *IFITM3*, *OAS1*, and *MX1*) expression in poly I:C-pretreated MDBK cells upon BVDV infection (Fig. 6G). These results indicate that the formation of the HK2-MAVS-VDAC1 complex induced by BVDV infection competitively disrupts the RIG-I–MAVS interaction, thereby inhibiting the RIG-I–MAVS signaling pathway-mediated IFN-β production.

### Glycolysis promotes BVDV replication

**Fig 7:**
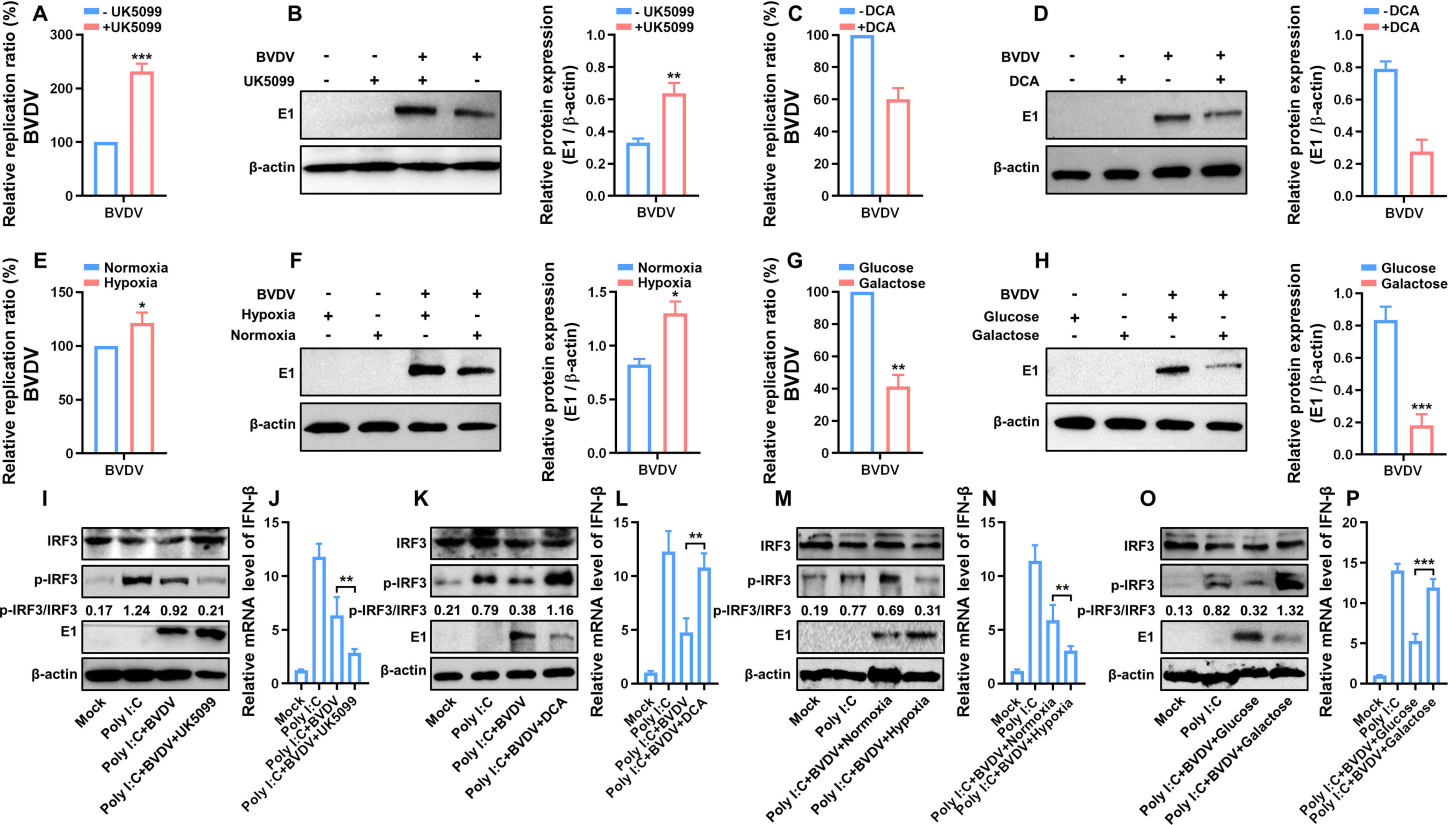
Glycolysis promotes BVDV replication. (**A**) BVDV replication in MDBK cells treated with the mitochondrial pyruvate transporter inhibitor UK5099, as measured by qRT-PCR. (**B**) Viral protein expression in UK5099-treated MDBK cells was detected by western blot. (**C**) BVDV replication in MDBK cells treated with the pyruvate dehydrogenase kinase inhibitor DCA, as assessed by qRT-PCR. (**D**) Viral protein expression in DCA-treated MDBK cells detected by western blot. (**E**) BVDV replication in MDBK cells cultured under normoxia (20% O_2_) or hypoxia (5% O_2_), measured by qRT-PCR. (**F**) Viral protein expression in MDBK cells under normoxia or hypoxia was detected by western blot. (**G**) BVDV replication in MDBK cells cultured under glucose (25 mmol/L) or galactose (25 mmol/L), as assessed by qRT-PCR. (**H**) Viral protein expression in MDBK cells cultured with glucose or galactose detected by western blot. (**I**) Levels of IRF3 and p-IRF3 in BVDV-infected MDBK cells pretreated with poly(I:C) and UK5099, detected by western blot. (**J**) IFN-β mRNA levels in BVDV-infected MDBK cells pretreated with poly(I:C) and UK5099, measured by qRT-PCR. (**K**) Levels of IRF3 and p-IRF3 in BVDV-infected MDBK cells pretreated with poly(I:C) and DCA, detected by western blot. (**L**) IFN-β mRNA levels in BVDV-infected MDBK cells pretreated with poly(I:C) and DCA, measured by qRT-PCR. (**M**) Levels of IRF3 and p-IRF3 in BVDV-infected MDBK cells pretreated with poly(I:C) under normoxia or hypoxia, detected by western blot. (**N**) IFN-β mRNA levels in BVDV-infected MDBK cells pretreated with poly(I:C) under normoxia or hypoxia, measured by qRT-PCR. (**O**) Levels of IRF3 and p-IRF3 in BVDV-infected MDBK cells pretreated with poly(I:C) under galactose or glucose culture conditions, detected by western blot. (**P**) IFN-β mRNA levels in BVDV-infected MDBK cells pretreated with poly(I:C) under galactose or glucose culture condition, measured by qRT-PCR. Data are representative of three independent experiments and are presented as mean ± SD. *P < 0.05, **P < 0.01, ***P < 0.001; ns, not significant.

To examine the impact of glycolysis induced by BVDV infection on viral replication, MDBK cells were treated with UK5099 (a pyruvate transporter inhibitor) and DCA (a pyruvate dehydrogenase kinase inhibitor) for 12 h prior to infection with BVDV (MOI = 5) for 48 h. The viral replication capacity was subsequently assessed, and the results indicated that treatment with UK5099 significantly enhanced BVDV replication, as determined by qRT-PCR (Fig. 7A) and western blot analysis (Fig. 7B). In contrast, viral RNA replication (Fig. 7C) and protein expression (Fig. 7D) were markedly inhibited in MDBK cells treated with DCA, suggesting that glycolysis facilitates BVDV replication. Additionally, we observed that viral RNA replication (Fig. 7E) and protein expression (Fig. 7F) were significantly elevated in BVDV-infected MDBK cells cultured under hypoxic conditions (5% O_2_) compared to those under normoxic conditions (20% O_2_). We also observed that both viral RNA replication (Fig. 7G) and protein expression (Fig. 7H) of BVDV were markedly suppressed when cells were cultured with galactose instead of glucose. These results indicate that an enhanced glycolytic flux promotes BVDV replication. To further investigate the mechanisms by which BVDV infection-induced glycolysis facilitates viral replication, MDBK cells were cultured under various conditions, including UK5099, DCA, normoxia, hypoxia, glucose, and galactose, for 12 h. The cells were then transfected with poly(I:C) for 24 h and infected with BVDV (MOI = 5) for 48 h. The levels of p-IRF3 and mRNA expression levels of IFN-β subjected to different treatments were analyzed. The results indicated that UK5099 treatment and hypoxic conditions markedly decreased p-IRF3 level (Fig. 7I and M), consequently inhibiting IFN-β expression (Fig. 7J and N). Treatment with DCA and galactose conditions significantly increased p-IRF3 level (Fig. 7K and O), thereby promoting IFN-β expression (Fig. 7L and P). These findings suggest that BVDV-induced glycolysis promotes viral replication via modulating IFN-β expression.

### Glycolysis-mediated lactate production favors BVDV replication via attenuating IFN-I responses

**Fig 8:**
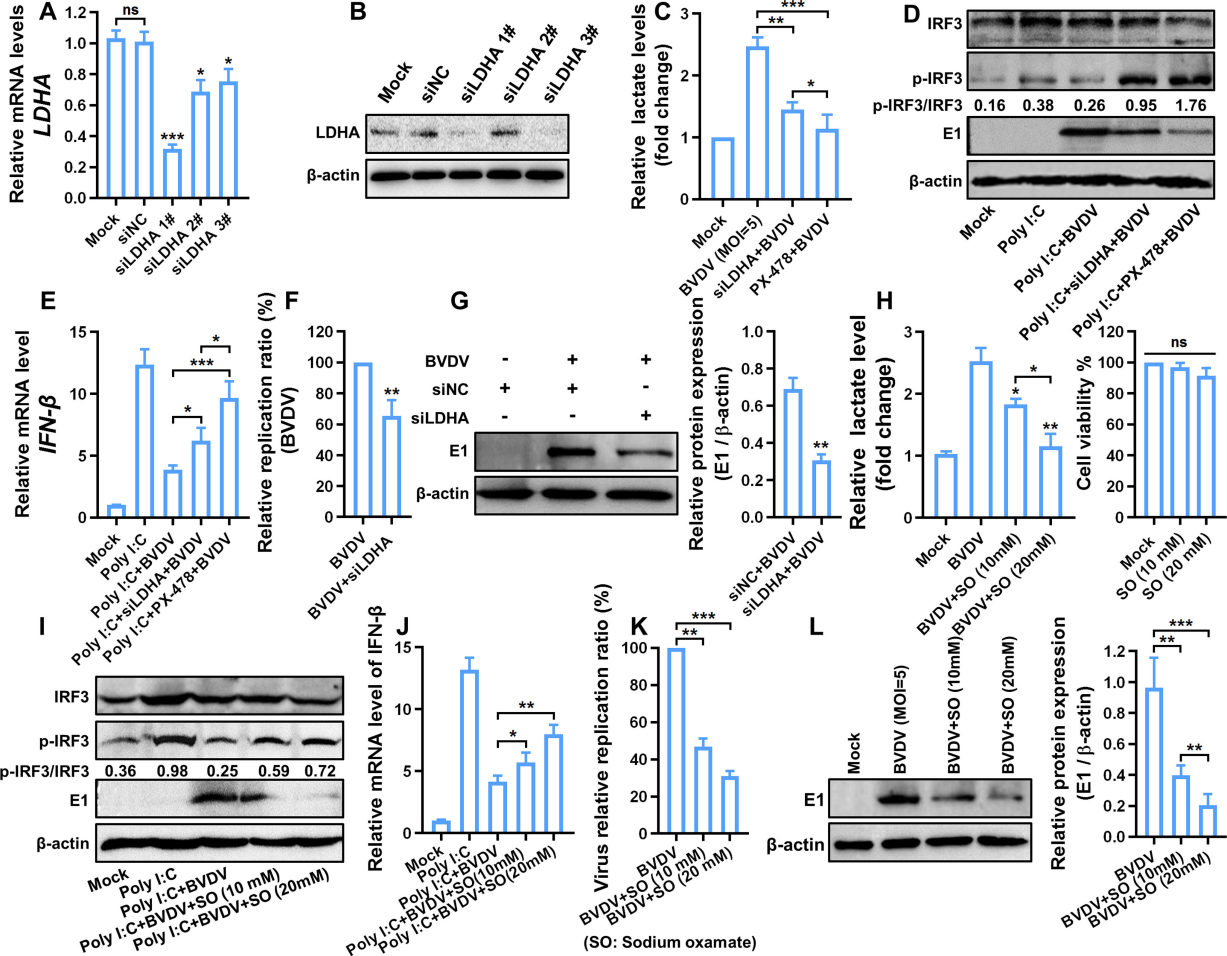
Glycolysis-derived lactate enhances BVDV replication by attenuating IFN-I responses. (**A**) Knockdown efficiency of siRNAs targeting LDHA evaluated by qRT-PCR. (**B**) Knockdown efficiency of siRNAs targeting LDHA assessed by western blot. (**C**) Lactate levels in BVDV-infected MDBK cells transfected with siLDHA 3# or treated with PX-478. (**D**) Levels of IRF3 and p-IRF3 in BVDV-infected MDBK cells pretreated with poly(I:C) and siLDHA or PX-478. (**E**) IFN-β mRNA levels in BVDV-infected MDBK cells pretreated with poly(I:C) in combination with siLDHA or PX-478. (**F**) BVDV replication in LDHA-knockdown MDBK cells measured by qRT-PCR. (**G**) Viral protein expression in LDHA-knockdown MDBK cells detected by western blot. (**H**) Lactate levels in MDBK cells treated with sodium oxamate (SO), an LDHA-specific inhibitor. (**I**) Levels of IRF3 and p-IRF3 in BVDV-infected MDBK cells pretreated with poly(I:C) and SO. (**J**) IFN-β mRNA levels in BVDV-infected MDBK cells pretreated with poly(I:C) and SO. (**K**) BVDV replication in SO-treated MDBK cells, as measured by qRT-PCR. (**L**) Viral protein expression in SO-treated MDBK cells was detected by western blot. Data are representative of three independent experiments and are presented as mean ± SD. *P < 0.05, **P < 0.01, ***P < 0.001; ns, not significant.

**Fig 9:**
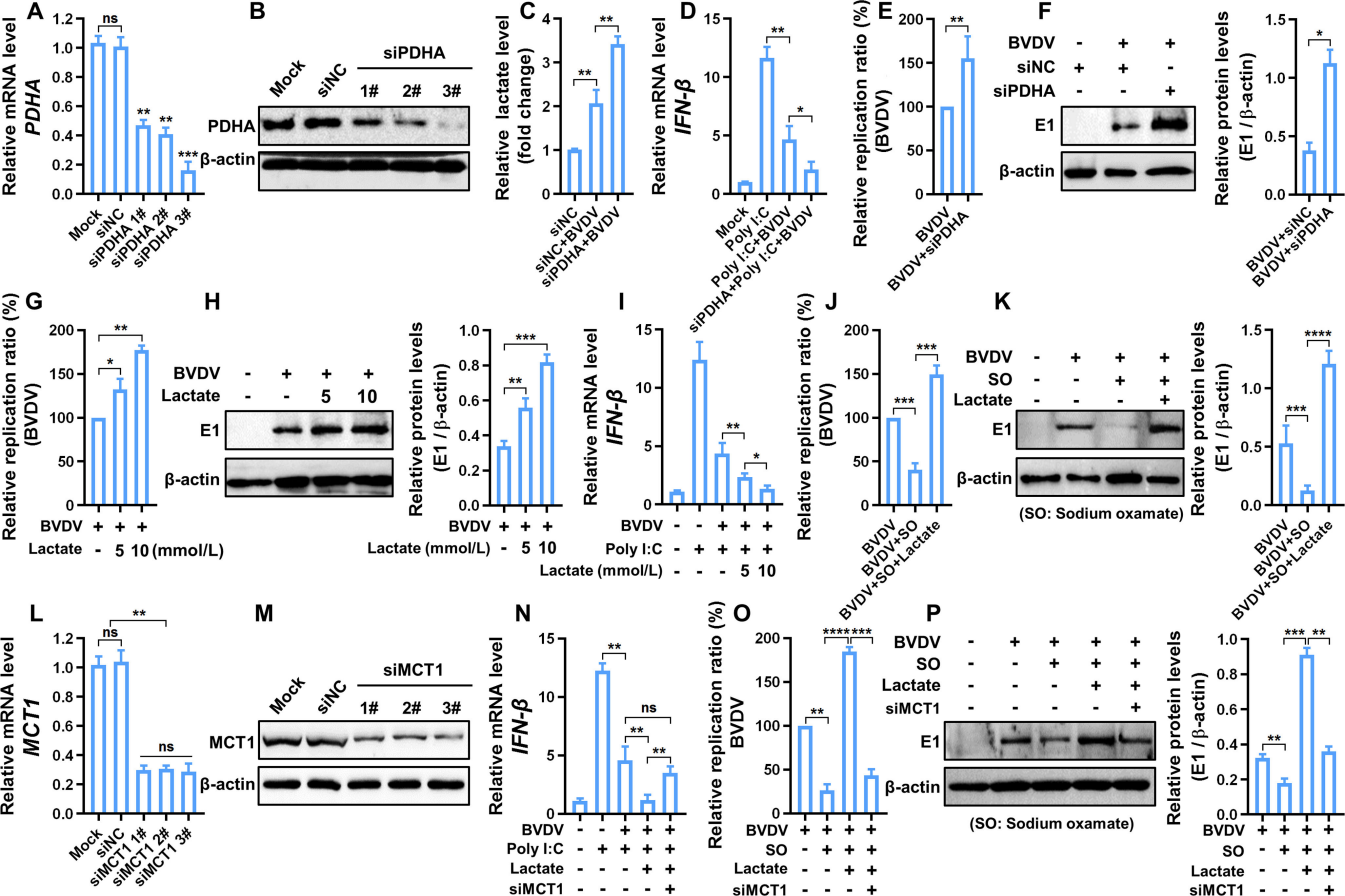
Inhibition of PDHA and lactate transporter MCT1 attenuates IFN-I response to promote viral infection. (**A**) Knockdown efficiency of siRNAs targeting PDHA evaluated by qRT-PCR. (**B**) Knockdown efficiency of siRNAs targeting PDHA detected by western blot. (**C**) Lactate levels in BVDV-infected MDBK cells transfected with siPDHA 3#. (**D**) IFN-β mRNA levels in BVDV-infected MDBK cells pretreated with poly(I:C) and siPDHA. (**E**) BVDV replication in PDHA-knockdown MDBK cells measured by qRT-PCR. (**F**) Viral protein expression in PDHA-knockdown MDBK cells was detected by western blot. (**G**) BVDV replication in MDBK cells supplemented with lactate, measured by qRT-PCR. (**H**) Viral protein expression in MDBK cells supplemented with lactate. (**I**) IFN-β mRNA levels in BVDV-infected MDBK cells treated with poly(I:C) and lactate. (**J**) BVDV replication in MDBK cells treated with sodium oxamate (SO) and lactate. (**K**) Viral protein expression in MDBK cells treated with SO and lactate. (**L**) Knockdown efficiency of siRNAs targeting MCT1 evaluated by qRT-PCR. (**M**) Knockdown efficiency of siRNAs targeting MCT1 detected by western blot. (**N**) IFN-β mRNA levels in BVDV-infected MDBK cells treated with poly(I:C), SO, and lactate, measured by qRT-PCR. (**O**) BVDV replication in MDBK cells treated with SO, lactate, and siMCT1, measured by qRT-PCR. (**P**) Viral protein expression in MDBK cells treated with SO, lactate, and siMCT1, detected by western blot. Data are representative of three independent experiments and are presented as mean ± SD. *P < 0.05, **P < 0.01, ***P < 0.001, and ****P < 0.0001; ns, not significant.

Glycolysis refers to anaerobic catabolism of glucose into pyruvate, which is subsequently converted into lactate by LDHA. To investigate the role of lactate in BVDV-induced glycolysis, which promotes viral replication, siRNAs targeting LDHA were designed, and their silencing efficiency was assessed using qRT-PCR (Fig. 8A) and western blot (Fig. 8B), and siLDHA 3# was selected for further investigation. Subsequently, lactate levels, IFN-I expression, and viral replication were measured in LDHA-knockdown MDBK cells following BVDV infection. The results showed that knockdown of LDHA significantly reduced lactate production (Fig. 8C) and increased the level of p-IRF3 (Fig. 8D), thereby enhancing IFN-β expression (Fig. 8E). Consequently, BVDV replication was strongly inhibited, as evidenced by a marked reduction in viral RNA replication (Fig. 8F) and protein expression (Fig. 8G). These findings indicate that silencing LDHA expression markedly enhances the IFN-I-mediated innate antiviral immune response, thereby inhibiting BVDV replication. Moreover, treatment with sodium oxamate (SO), a glycolysis inhibitor, led to a significant decrease in lactate production (Fig. 8H), accompanied by reduced levels of p-IRF3 (Fig. 8I) and IFN-β expression (Fig. 8J) in BVDV-infected MDBK cells, with effects showing dose dependency. Notably, SO treatment strongly inhibited BVDV replication, as reflected by substantial reductions in both viral RNA replication (Fig. 8K) and protein expression levels (Fig. 8L). Pyruvate dehydrogenase A (PDHA), a critical subunit of the pyruvate dehydrogenase complex, facilitates the oxidative decarboxylation of pyruvate to acetyl-CoA, thereby linking glycolysis to the tricarboxylic acid cycle. Following PDHA knockdown using siRNA (siPDHA 3#), with silencing efficiency verified by qRT-PCR (Fig. 9A) and western blot (Fig. 9B), we observed a marked increase in lactate levels in PDHA-knockdown MDBK cells post-BVDV infection (Fig. 9C), while IFN-β expression was significantly reduced (Fig. 9D). Additionally, PDHA knockdown markedly enhanced BVDV replication, as indicated by elevated viral RNA levels (Fig. 9E) and increased viral protein expression (Fig. 9F). Taken together, these results elucidate that lactate production derived from glycolysis facilitates BVDV replication by suppressing the IFN-I-mediated antiviral immune response.

### MCT1-dependent lactate transport attenuates IFN-I response to facilitate BVDV infection

We next examined the effect of exogenous lactate supplementation on BVDV replication. The results showed that lactate treatment significantly enhanced viral replication in infected MDBK cells in a dose-dependent manner, as demonstrated by increased viral RNA (Fig. 9G) and protein levels (Fig. 9H). Conversely, the lactate supplementation led to a dose-dependent reduction in IFN-β mRNA expression (Fig. 9I). To further validate the role of lactate, we inhibited glycolysis with SO and then supplied exogenous lactate to BVDV-infected MDBK cells. This rescue experiment revealed that lactate supplementation significantly alleviated the antiviral effect of SO, restoring BVDV replication at both the viral RNA (Fig. 9J) and protein levels (Fig. 9K). Monocarboxylate transporter 1 (MCT1) is critically involved in lactate transport across cell membranes. To explore its functional role, we designed specific siRNAs targeting MCT1 and evaluated their silencing efficacy using qRT-PCR (Fig. 9L) and western blot (Fig. 9M). Knockdown of MCT1 with siRNA (siMCT1 3#) significantly inhibited the intracellular uptake of exogenously supplied lactate in polyI:C-transfected MDBK cells infected with BVDV and supplemented with lactate (siMCT1+polyI:C+BVDV + lactate group). This inhibition led to a marked increase in IFN-β expression (Fig. 9N) compared to the control group (polyI:C+BVDV + lactate group). Furthermore, MCT1 knockdown strongly suppressed BVDV replication, as evidenced by qRT-PCR (Fig. 9O) and western blot (Fig. 9P). Together, these results highlight the importance of lactate transmembrane transport in facilitating BVDV infection through attenuating IFN-I-mediated antiviral immune response.

### Lactate is responsible for glycolysis-mediated RLR signaling inhibition by directly binding to MAVS

**Fig 10:**
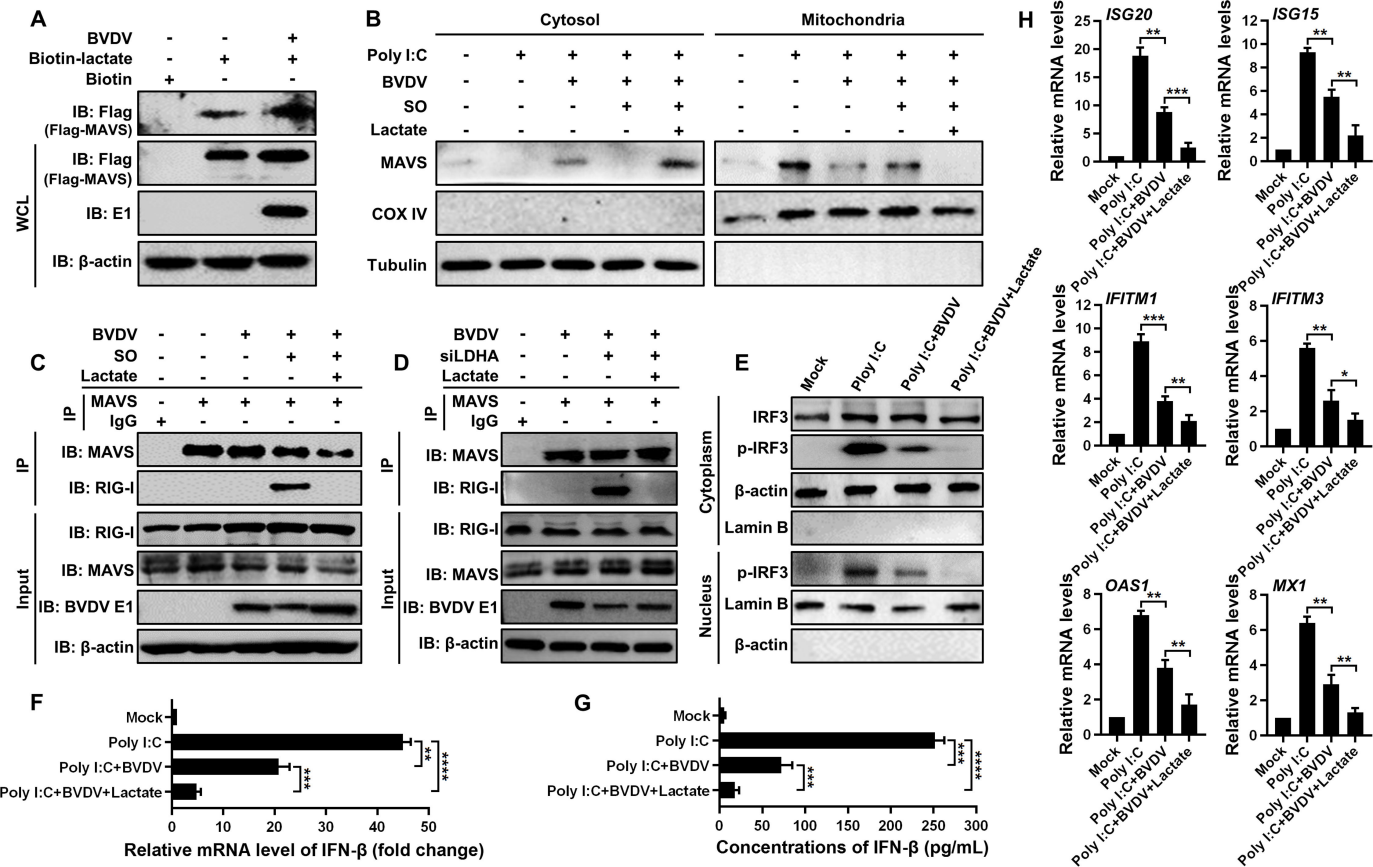
Lactate suppressed the RLR signaling pathway by directly binding to MAVS. (**A**) Interaction between lactate and MAVS assessed by biotin-labeled lactate pull-down assay. (**B**) Lactate-induced translocation of MAVS from mitochondria to cytoplasm, as detected by western blot. (**C**) Co-IP analysis of the endogenous RIG-I–MAVS interaction in SO-treated MDBK cells upon BVDV infection, with or without exogenous lactate supplementation. (**D**) Co-IP analysis of the endogenous RIG-I–MAVS interaction in LDHA-knockdown cells upon BVDV infection, with or without adding exogenous lactate supplementation. (**E**) Western blot analysis of IRF3 phosphorylation and nuclear translocation following lactate treatment. (**F–G**) IFN-β mRNA expression (**F**) and protein secretion (**G**) in poly I:C-pretreated MDBK cells upon BVDV infection, with exogenous lactate supplementation. (**H**) Expression levels of interferon-stimulated genes (*ISG20*, *ISG15*, *IFITM1*, *IFITM3*, *OAS1*, and *MX1*) under the same conditions as in panel F. Data are representative of three independent experiments and are presented as mean ± SD. *P < 0.05, **P < 0.01, ***P < 0.001, and ****P < 0.0001; ns, not significant.

To elucidate the underlying mechanism through which lactate suppresses IFN-I production, a biotin-labeled lactate pull-down assay was performed. The results revealed a direct interaction between lactate and MAVS (Fig. 10A), indicating that lactate may disrupt the RIG-I–MAVS complex and thereby inhibit IFN-I signaling. Furthermore, lactate was found to alter the subcellular distribution of MAVS, promoting its translocation from mitochondria to the cytoplasm. Inhibition of glycolysis-derived lactate production with sodium oxamate (SO) effectively blocked BVDV-induced cytoplasmic translocation of MAVS (Fig. 10B). Additionally, treatment with SO, a well-characterized LDHA inhibitor, significantly enhanced the endogenous RIG-I–MAVS interaction in BVDV-infected MDBK cells, whereas exogenous lactate supplementation impaired this association (Fig. 10C). To exclude potential off-target effects of SO and further validate these findings, we performed siRNA-mediated knockdown of LDHA in BVDV-infected cells. As shown in Fig. 10D, silencing LDHA markedly enhanced endogenous RIG-I–MAVS interaction, consistent with the results obtained using SO treatment. Notably, this enhanced interaction was reversed by the addition of exogenous lactate to LDHA-knockdown cells. Consistent with these observations, lactate-mediated disruption of the RIG-I–MAVS interaction led to suppressed phosphorylation and nuclear translocation of IRF3 (Fig. 10E). We further observed that exogenous lactate supplementation significantly reduced IFN-β mRNA expression (Fig. 10F) and protein secretion (Fig. 10G), as well as the expression of multiple interferon-stimulated genes, including *ISG20*, *ISG15*, *IFITM1*, *IFITM3*, *OAS1*, and *MX1* (Fig. 10H). Together, these results indicate that lactate acts as a key glucose metabolite that attenuates the RLR-MAVS signaling pathway. This inhibition occurs through the regulation of MAVS translocation by directly binding MAVS and modulating its translocation, ultimately impairing the antiviral immune response.

## DISCUSSION

Interferons (IFNs), especially type I IFNs, serve as the first line of defense against viral infections. Recent studies have underscored the crucial role of cellular energy metabolism in regulating innate immune responses, revealing a complex crosstalk between IFN signaling and metabolic reprogramming (45–47). Upon viral infection, virus-induced immune activation drives significant metabolic changes. Although these adaptations are generally aimed at supporting immune responses, they can also paradoxically suppress certain aspects of immunity, thereby establishing an intricate feedback loop (48–50). Additionally, cells upregulate catabolic processes to meet the heightened energy demands required for immune activation, further complicating the metabolic landscape (51, 52). Viruses such as the hepatitis C virus (HCV) and hepatitis B virus (HBV) exploit host energy metabolism to secure ample energy and biosynthetic precursors essential for viral replication (53, 54).

Similar to other viruses, BVDV exploits the host cell’s metabolic machinery to produce essential biomolecules and energy required for viral replication. In this study, we observed that BVDV infection significantly increased lactate levels both *in vivo* and *in vitro*, indicating the induction of glucose metabolic reprogramming; however, the underlying mechanisms and biological implications remain unclear. Previous studies have shown that viruses such as Epstein-Barr virus, Senecavirus A, and respiratory syncytial virus can trigger metabolic reprogramming resembling the “Warburg effect”—shifting energy production from oxidative phosphorylation to glycolysis (42, 44, 55). This transition is frequently associated with upregulation and activation of the HIF-1α (44). Under normoxic conditions, HIF-1α is rapidly hydroxylated by prolyl hydroxylases and degraded via the ubiquitin-proteasome pathway (56). Hypoxia, however, inhibits hydroxylation, leading to HIF-1α stabilization and nuclear translocation. In the nucleus, HIF-1α dimerizes with HIF-1β and binds to hypoxia-response elements (HREs), promoting the transcription of key glycolytic genes including *GLUT1*, *HK2*, *PFKP*, and *LDHA*, which collectively enhance glucose uptake and glycolytic flux. It is indicated that ROS contributes to HIF-1α stabilization and activation (44). Following BVDV infection, we observed significant upregulation of phosphorylated eIF2α—a downstream target of PERK—as well as increased expression of the PERK effectors ATF4 and GADD34. Inhibition of ER stress markedly suppressed the expression of oxidative stress-related genes of *HMOX-1*, *TXN*, and *PRDX-6*, indicating impaired ROS scavenging capacity and consequent ROS accumulation. Notably, although PERK-mediated eIF2α phosphorylation typically induces global translational arrest as part of the integrated stress response, BVDV appears to circumvent this blockade. Specifically, the upregulated GADD34, a regulatory subunit of protein phosphatase 1, promotes dephosphorylation of eIF2α, thereby selectively restoring translation of stress-adaptive mRNAs, such as those encoding HIF-1α and key glycolytic enzymes, while global protein synthesis remains suppressed (57–59). This selective translational recovery allows BVDV to sustain HIF-1α-driven glycolytic reprogramming despite the eIF2α-mediated translational blockade. Thus, BVDV effectively converts a host defense mechanism into a pro-viral signal, supporting its replication while evading innate immunity. In line with this, a recent study demonstrated that BVDV infection activates ROS-endoplasmic reticulum (ER) stress axis via the PERK pathway to induce autophagy, which, in turn, inhibits IFN-I signaling and promotes viral replication (22). Strikingly, this strategy contrasts with that of other viruses such as porcine hemagglutinating encephalomyelitis virus (PHEV) and transmissible gastroenteritis virus (TGEV), where PERK activation restricts viral replication (60, 61), highlighting the virus-specific outcomes of ER stress engagement. In this work, we observed that BVDV infection markedly decreased HIF-1α hydroxylation, thereby promoting its stabilization, upregulated expression, and nuclear translocation. Treatment with the HIF-1α inhibitor PX-478 significantly suppressed the expression of glycolytic genes (*GLUT1*, *HK2*, *PFKP*, and *LDHA*), leading to reduced glucose uptake and lactate production, alongside increased IFN-β levels. Importantly, we demonstrated that HIF-1α stabilization and activation in infected cells depend on BVDV-induced ROS production. Inhibition of ROS with NAC significantly elevated hydroxylated HIF-1α levels and suppressed the expression of glycolysis-related genes. In conclusion, our results indicate that BVDV infection enhances HIF-1α-mediated glycolysis to facilitate viral replication.

The selection of HK2 as the focus of this study was guided by its unique role in linking glycolysis to innate immune regulation. Among the four mammalian hexokinase isoforms, HK2 is the most catalytically active and is selectively upregulated under cellular stress conditions, including viral infection (62–64). Importantly, emerging evidence has directly implicated HK2 in the modulation of antiviral immunity. Seminal studies have demonstrated that HK2 interacts with MAVS, competing with RIG-I for MAVS binding and thereby suppressing IFN-I production (40–42). This mechanism has been validated in multiple viral infection models, including Senecavirus A (42), and aligns closely with our observations in BVDV-infected cells. For other glycolytic enzymes, we applied a similar rationale—selecting isoforms predominantly expressed in our bovine cell system and functionally implicated in metabolic reprogramming during infection (e.g., PFKP among PFK isoforms, LDHA over LDHB for lactate production). Furthermore, HIF-1α is a well-established transcriptional activator of pro-inflammatory cytokines, including IL-1β and CXCL8 (65, 66). This raises an apparent paradox: if BVDV stabilizes and activates HIF-1α, how does it avoid triggering a detrimental inflammatory response? Our findings suggest that BVDV resolves this dilemma by temporally and functionally decoupling HIF-1α’s metabolic and immune functions. Following infection, HIF-1α is stabilized and translocates to the nucleus, where it preferentially drives the expression of glycolytic genes (GLUT1, HK2, PFKP, and LDHA) rather than inflammatory mediators. This results in enhanced glycolytic flux and marked lactate accumulation. Notably, IFN-β expression peaks early post-infection but declines sharply as lactate levels rise, indicating that the antiviral response is initiated but subsequently overridden by metabolic reprogramming. Mechanistically, BVDV-induced glycolysis promotes the formation of an HK2/MAVS/VDAC1 complex, which disrupts RIG-I–MAVS interaction and impairs downstream signaling. Concurrently, lactate—the end product of enhanced glycolysis—directly binds to MAVS and alters its mitochondrial localization, further suppressing the RIG-I–MAVS signaling axis. Thus, the very products of HIF-1α-driven glycolysis—HK2 and lactate—serve as viral effectors to dismantle innate immunity. This strategy, also observed in Senecavirus A infection (42), allows BVDV to harness HIF-1α’s metabolic benefits while evading its pro-inflammatory consequences. Consistent with these findings, we observed a marked increase in lactate production following BVDV infection, a hallmark of enhanced anaerobic glycolysis. This metabolic reprogramming not only supports viral replication by supplying ATP but also actively interferes with antiviral immune responses. Previous studies have shown that lactate can directly bind to MAVS and inhibit RLR signaling, thereby raising the threshold for innate immune activation and limiting IFN-I production (67). Our results corroborate these reports and further demonstrate that BVDV-induced glycolytic activation suppresses RLR-dependent IFN-β expression specifically via lactate-mediated impairment of RIG-I–MAVS complex formation. These findings collectively reveal a dual role for HIF-1α-driven glycolysis in BVDV infection: providing metabolic support for viral replication while simultaneously neutralizing the host’s antiviral defenses.

From an evolutionary perspective, the multistep cascade we describe—ER stress, HIF-1α stabilization, glycolysis, and lactate accumulation—likely represents a conserved host adaptive response designed to balance antiviral immunity against immune-mediated tissue damage. Glycolysis provides rapid ATP and biosynthetic precursors to meet the heightened energy demands of infected cells (32), while lactate, as its end product, directly binds MAVS and dampens RLR signaling (40, 67), thereby serving as a physiological “brake” that prevents excessive inflammation. However, BVDV appears to have hijacked this host feedback loop. By actively inducing ER stress and ROS production, the virus drives sustained HIF-1α activation and hyperglycolysis, resulting in excessive and prolonged lactate accumulation. This high-lactate environment chronically suppresses MAVS-mediated IFN-I responses, converting the host’s self-limiting mechanism into a viral immune evasion strategy. Simultaneously, enhanced glycolysis supplies the energy and precursors necessary for efficient viral replication. This dual exploitation—metabolic support and immune suppression—underscores how BVDV has adapted to thrive within the host’s own regulatory framework.

While traditionally regarded merely as a byproduct of anaerobic glycolysis, lactate is now acknowledged as a multifunctional molecule involved in diverse cellular processes. Recent studies have revealed its participation in a wide array of biological functions, including ATP generation in cancer cells, modulation of inflammatory responses, involvement in post-translational modifications, and regulation of immune processes such as macrophage polarization, T-helper cell differentiation, and tumor immune surveillance (68–71). In the context of BVDV infection, our previous work demonstrated that the virus significantly upregulates the expression of key glycolytic enzymes, including HK2, PK, and LDHA, at both transcriptional and protein levels, leading to enhanced lactate production both *in vivo* and *in vitro* (43). Despite these findings, the mechanisms through which BVDV exploits glycometabolic reprogramming to facilitate viral replication remain poorly understood. In this study, we aimed to elucidate how BVDV modulates host glycolysis to facilitate its replication. We found that BVDV infection promotes the formation of a ternary complex consisting of HK2, MAVS, and VDAC1 (designated the HK2-MAVS-VDAC1 complex), which disrupts the interaction between RIG-I and MAVS and consequently inhibits the RIG-I–MAVS signaling pathway. HK2, a pivotal glycolytic enzyme, catalyzes the first step of glycolysis by converting glucose to glucose-6-phosphate. VDAC1, a mitochondrial porin located on the outer mitochondrial membrane, serves as the primary receptor for hexokinase and plays an essential role in metabolic regulation. The identification of BVDV-induced HK2-MAVS-VDAC1 complex formation unveils a novel mechanism by which the virus concurrently manipulates host glycolytic flux and mitochondrial antiviral signaling to evade innate immune responses.

Furthermore, LDHA, a key enzyme in the glycolytic pathway, preferentially catalyzes the conversion of pyruvate to lactate—a central process in virus-induced glycometabolic reprogramming (72, 73). In this study, we found that BVDV infection significantly upregulates LDHA expression, thereby enhancing LDHA-dependent lactate production. We further demonstrated that lactate alters the subcellular localization of MAVS, promoting its translocation from mitochondria to the cytoplasm. Lactate was also shown to interact directly with MAVS, disrupting the association between RIG-I and MAVS and consequently inhibiting the RLR-mediated IFN signaling pathway. These results illustrate how BVDV subverts innate immune signaling by elevating lactate production and perturbing MAVS localization. Although previous studies have suggested that targeting LDHA may inhibit tumor growth and replication (74, 75), its role in viral infection remains poorly characterized. Here, we show that pharmacological inhibition of LDHA effectively suppresses BVDV replication, inhibiting its potential as a novel antiviral strategy. Moreover, the identification of metabolic enzymes and transporters as critical facilitators of viral replication opens new avenues for antiviral strategies. For instance, HK2 inhibitors, including 2-deoxyglucose, dichloroacetate, and 3-bromopyruvate (76–78), may serve as promising candidates against BVDV infection. Additionally, monocarboxylate transporters (MCTs), especially MCT1, which mediate lactate shuttling across membranes (79), represent another target. Our data indicate that knockdown of MCT1 restrains viral replication by blocking lactate transport, thereby potentiating the IFN-I-mediated antiviral immune response. Thus, inhibiting MCT1 may confer antiviral effects against BVDV. In summary, targeting virus-altered metabolic pathways may improve immune-based therapies and provide new directions for antiviral drug development.

**Fig 11:**
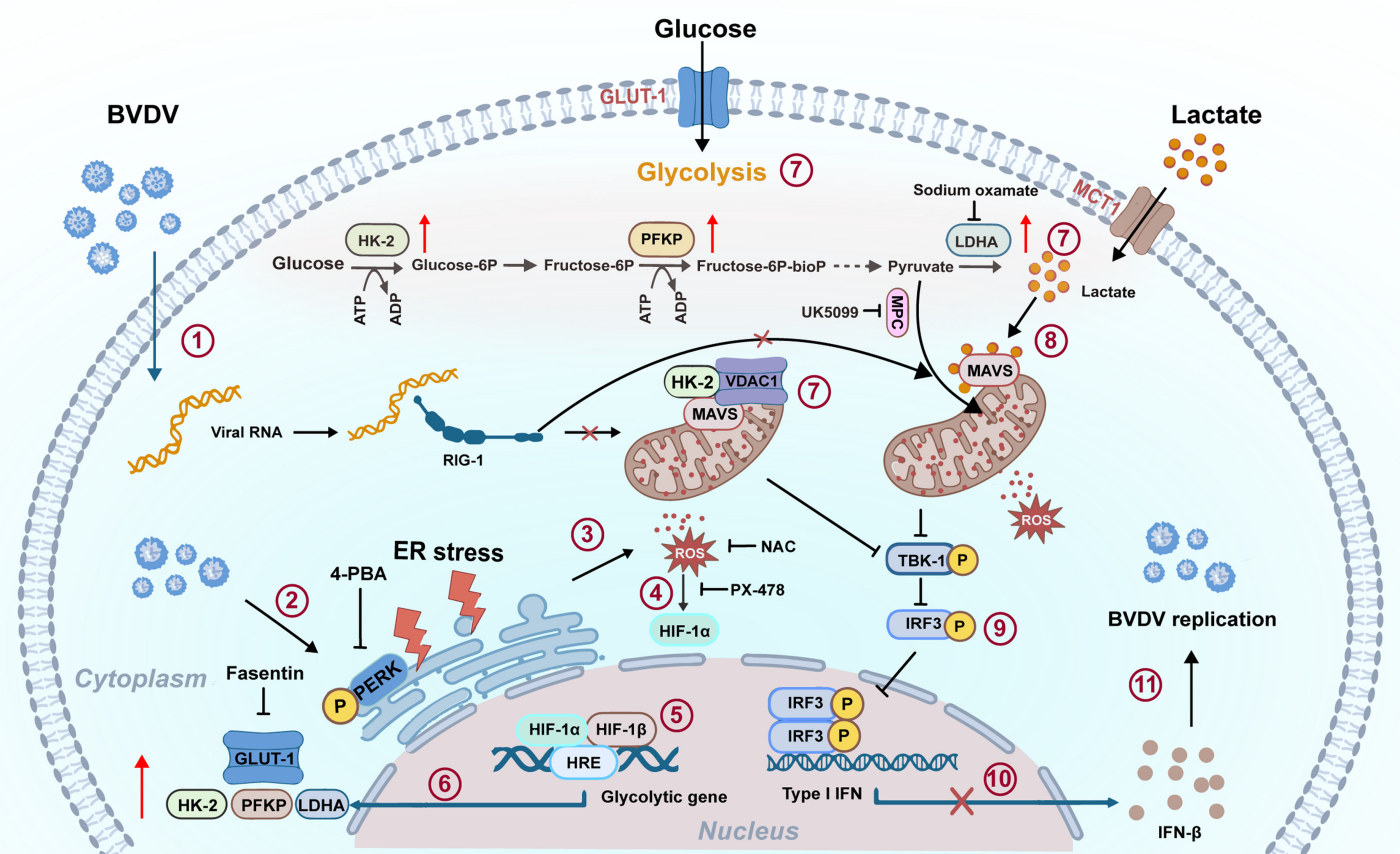
Schematic illustration of the mechanism by which BVDV infection inhibits the RIG-I/MAVS-mediated IFN-I production via the ROS–HIF-1α–glycolysis axis.

In conclusion, this study demonstrates that BVDV infection facilitates its replication by subverting RIG-I/MAVS signaling pathway and inhibiting IFN-I production through ROS–HIF-1α axis-driven glycometabolic reprogramming. A schematic summary of this study was presented in Fig. 11. We show that BVDV infection promotes the assembly of an HK2/MAVS/VDAC1 complex, which interferes with the interaction between RIG-I and MAVS. In parallel, BVDV infection enhances glycolysis-derived lactate production, which competitively binds MAVS and impairs its mitochondrial localization, thereby further suppressing the RIG-I/MAVS-mediated IFN-I response. These findings provide novel mechanisms and insights into how BVDV infection reprograms host metabolism to evade antiviral immunity and support its replication. It should be noted that the present study did not employ isotope-labeled glucose tracing, which is considered a gold standard approach for quantitatively assessing glycolytic flux and metabolite routing. Future studies utilizing such methodology could provide additional depth and validation for the metabolic reprogramming events described here.

## MATERIALS AND METHODS

### Cells, viruses, and plasmids

Madin-Darby bovine kidney (MDBK) cells and primary bovine testicular (BT) cells were cultured in high-glucose Dulbecco’s modified Eagle’s medium (DMEM; Gibco, USA) supplemented with 10% fetal bovine serum (FBS; Tianhang, China) at 37°C under 5% CO2. The BVDV strain AV69 was propagated in both cell types. For infection experiments, cells at ≥80% confluency were infected with BVDV at a multiplicity of infection (MOI) of 5. Viral titers were determined using the 50% tissue culture infective dose (TCID_50_) assay, as previously described (23). Additionally, recombinant eukaryotic expression plasmids—pCMV^Myc^-HK2, pCMV^3×Flag^-MAVS, pCMV^Myc^-VDAC1, and pCMV^HA^-RIG-I—were constructed to express bovine HK2, MAVS, VDAC1, and RIG-I, respectively. Briefly, the full-length coding sequences of bovine *HK2* (XM_015473383.2), *MAVS* (NM_001046620.2), *VDAC1* (NM_174485.4), and *RIG-I* (XM_024996055.1) were amplified by RT-PCR from total RNA extracted from BVDV-infected MDBK cells, using gene-specific primers (Table S1).

### Antibodies

The following antibodies were obtained from commercial sources. Rabbit anti-HK2 antibody (bs-3993P), anti-RIG-I antibody (bsm-61573R), anti-VDAC1 antibody (bs-1461R), and anti-IFNAR antibody (bs-4116R) were purchased from Beijing Bioss Biotechnology Company (China). Rabbit anti-GLUT1 (AF5462), anti-PFKP (DF3234), anti-Lamin B (AF5161), and mouse anti-HIF1α (BF8002) were from Affinity Biosciences (USA). Rabbit anti-IgG antibody (AC005), anti-PDHA antibody (A1895), anti-LDHA antibody (A1146), anti-MCT1 antibody (A3013), anti-MAVS antibody (A5764), and mouse anti-IgG antibody (AC011) were acquired from Wuhan Aibotek Biotechnology (China). Rabbit anti-Hydroxy-HIF1α antibody (#3434), anti-HA antibody (#3724), anti-β-actin antibody (#4970), anti-TBK1 antibody (#38066), anti-eIF2α antibody (#9079), anti-phospho-eIF2α (Ser51) antibody (#3398), and mouse anti-tubulin antibody (#86298), anti-COX IV antibody (#11967) were purchased from Cell Signaling Technology (USA). Rabbit anti-GAPDH antibody (48102) was supplied from Hangzhou Bioker Biotechnology (China). Rabbit anti-p-TBK1 antibody (ab109272), anti-IRF3 antibody (ab68481), anti-p-IRF3 antibody (ab76493), and goat anti-mouse IgG antibody (FITC) (ab6785) were from Abcam (UK). Rabbit anti-Myc antibody (C3956) was from Sigma (USA). Rabbit anti-Flag antibody (AF0036) was from Shanghai Beyotime Biotechnology (China). Goat anti-mouse IgG HRP-link (115-035-003), goat anti-rabbit IgG HRP-link (111-035-144), and horseradish peroxidase-conjugated IgG fraction monoclonal mouse anti-rabbit IgG (light chain specific) (211-032-171) were obtained from Jackson ImmunoResearch (USA). Rabbit anti-ATF6 antibody (A0202), anti-BiP/GRP78 antibody (A0241), anti-phospho-IRE1-S724 antibody (AP1146), anti-PERK antibody (AP1146), anti-ATF4 antibody (A7237), and anti-GADD34/PPP1R15A (A16260) were from Abclonal (China). Mouse anti-BVDV E1 and NS5B antibodies were prepared by our laboratory.

### Glucose uptake assay

The uptake of glucose in MDBK cells infected with BVDV was evaluated using a 2-NBDG Glucose Uptake Assay Kit (fluorescent; ab287845, Abcam, UK), according to the manufacturer’s instructions. Mock-infected MDBK cells cultured in a 96-well plate were used as controls. MDBK cells were infected with BVDV at an MOI of 5, either in the absence or presence of inhibitors (PX-478 or Fasentin), for 24 h. After infection, the supernatant was aspirated, and the cells were incubated with 100 µM 2-NBDG staining solution for 20 min. Following one wash step, intracellular fluorescence was measured using a flow cytometer equipped with the FITC channel (BD, USA). The experiment was performed with three independent biological replicates, each including three technical replicates.

### Analysis of glucose metabolism in BVDV-infected cells

In this study, the effect of BVDV infection on glucose metabolism was assessed by quantifying multiple glucose metabolites using commercially available kits. The metabolites analyzed included glucose, pyruvate, succinate, phosphoenolpyruvic acid (PEP), oxaloacetate, dihydroxyacetone phosphate (DHAP), aconitate, glucose-1-phosphate (G1P), fumaric acid, malate, 2,3-bisphosphoglycerate (2,3-DPG), maleic acid, andα-ketoglutarate. Kits from Abcam (UK), LSM Bio (China), QIYAO (China), and Cell Biolabs (USA) were used according to the manufacturer’s instructions. Briefly, MDBK cells were seeded in 6-well plates and allowed to reach approximately 80% confluency before infection with BVDV (MOI = 5) for 48 h. After infection, cells were harvested, and the metabolite levels were measured as per the kit protocols. The experiment included three independent biological replicates, each with three technical replicates.

### Glycolytic activity analysis in BVDV-infected cells

The extracellular acidification rate (ECAR) was measured to assess glycolytic activity in MDBK cells infected with BVDV, using a Seahorse XFe96 Analyzer (Agilent, USA) as described previously (40). Mock-infected MDBK cells cultured in a Seahorse 96-well plate served as controls. Cells at approximately 90% confluency were infected with BVDV (MOI = 5) for 12 h and 24 h. After infection, the cells were washed once with Seahorse XF assay medium (pH 7.4) and then incubated in the same medium supplemented with 2 mM glutamine for 60 min. Glucose (10 mM; Solarbio, China), oligomycin (1 µM; TargetMol, USA), and 2-deoxy-D-glucose (2-DG, 50 mM; TargetMol, USA) were added to ports A, B, and C of the hydrate cartridge, respectively. Data were analyzed following the completion of the Seahorse XFe96 run. Three independent biological replicates, each with three technical replicates, were performed.

### Measurement of intracellular ATP level in BVDV-infected cells

MDBK cells were cultured to approximately 80% confluency in either high-glucose (4.5 g/L) or low glucose (1.0 g/L) DMEM (Solarbio, China) supplemented with 10% FBS. The cells were then infected with BVDV (MOI = 5) for 48 h. After infection, cells were harvested and intracellular ATP levels were measured using an ATP Assay Kit (ab83355; Abcam, UK) according to the manufacturer’s instructions. The experiment was performed with three independent biological replicates, each including three technical replicates.

### Determination of lactate levels *in vivo* and *in vitro*

Healthy 5-month-old calves (*n* = 6) that were confirmed to be free of BVDV antigens and obtained from a local cattle farm were divided into two groups: a mock-infected control group and a BVDV-infected experimental group. Calves in the infected group were exposed to BVDV according to an established protocol (80, 81) with minor modifications. Specifically, each calf in the BVDV-infected group received 5 mL of BVDV suspension (10^5^ TCID_50_) intranasally (2.5 mL per naris) along with 1 mL orally. Serum samples were collected from the calves in each group at various time points, and lactate concentration was measured using the Lactate Assay Kit (MAK064, Sigma-Aldrich, USA) in accordance with the manufacturer’s instructions. For *in vitro* assessment of lactate levels, MDBK cells were cultured in 6-well plates until reaching over 80% confluency and then infected with BVDV (MOI = 5). Samples were collected at different time points post-infection, and lactate concentrations were measured using the same Lactate Assay Kit. The experiment included three independent biological replicates, each with three technical replicates.

### Quantitative reverse-transcription PCR (qRT-PCR)

Total RNA was extracted from tissues of BVDV-infected calves and from MDBK cells under different experimental conditions using the FastPure Cell/Tissue Total RNA Isolation Kit V2 (RC112-01; Vazyme, China) according to the manufacturer’s instructions. First-strand cDNA was then synthesized using the BeyoRT II First-Strand cDNA Synthesis Kit with gDNA Eraser (D7170S; Beyotime, China). The relative expression levels of target genes were determined by quantitative real-time PCR with *β-actin* as the internal reference gene, using the LightCycler 480 SYBR Green I (4887352001; Roche, Germany) on a QuantStudio 5 Real-Time PCR system (Applied Biosystems, USA). The primers used for qRT-PCR are listed in Table S1. All experiments were performed with three independent biological replicates, each consisting of three technical replicates.

### Membrane protein extraction

Total cellular membrane proteins were extracted using a Membrane Protein Extraction Kit (C500049; Sangon, China) in accordance with the manufacturer’s instructions. Briefly, MDBK cells were harvested at 0, 12, 24, and 48 h post-infection with BVDV (MOI = 5). The cells were washed three times with pre-cooled washing buffer and then incubated with an extraction buffer (supplemented with protease inhibitor phenylmethylsulfonyl fluoride [PMSF] and dithiothreitol as provided in the kit) at 4°C for 20 min, followed by ultrasonication 3–4 times on ice. The lysates were centrifuged at 14,000 × *g* for 30 min at 4°C. The membrane protein-containing lower layer was collected for subsequent western blot analysis.

### Nuclear and cytoplasmic protein extraction

Cytoplasmic and nuclear proteins were extracted using a Nuclear and Cytoplasmic Protein Extraction Kit (20126ES; Yeasen, China) according to the manufacturer’s instructions. Briefly, MDBK/BT cells were harvested at 0, 12, 24, and 48 h post-infection with BVDV (MOI = 5). The cells were incubated with Cytosolic Protein Extraction Reagent A supplemented with 1 mM PMSF for 15 min at 4°C. Cytosolic Protein Extraction Reagent B was then added, and the mixture was vortexed for 10 s and incubated at 4°C for 60 s. The cytosolic fraction was collected by centrifugation at 16,000 × *g* for 10 min at 4°C. The remaining pellet was resuspended in Nuclear Protein Extraction Reagent C, vortexed for 10 s, and incubated at 4°C for 20 min. Nuclear proteins were isolated by centrifugation at 16,000 × *g* for 10 min at 4°C. Both cytoplasmic and nuclear protein extracts were subsequently analyzed by western blot.

### Western blot analysis

MDBK/BT cells under different treatments were harvested, and total cellular proteins were extracted using RIPA Buffer (#9806; Cell Signaling Technology, USA). Protein concentrations were determined with the Bicinchoninic Acid (BCA) Protein Assay Kit (Thermo Scientific Pierce, USA) according to the manufacturer’s instructions. Equal amounts of total protein were separated by 10%–15% sodium dodecyl sulfate-polyacrylamide gel electrophoresis (SDS-PAGE) gels and transferred onto polyvinylidene difluoride (PVDF) membranes (Vazyme, China) using the semi-dry transfer system. The membranes were blocked with 5% skim milk and then incubated with appropriate primary and secondary antibodies. Protein bands were detected using the BeyoECL Star (P0018AM; Beyotime, China) in accordance with the manufacturer’s instructions. Western blot analyses were performed in replicates to ensure technical reproducibility.

### Immunofluorescence assay

In summary, following various treatments, cell samples in 12-well plates were washed three times with phosphate-buffered saline (PBS) and fixed with 4% paraformaldehyde for 30 min. After washing, the cells were permeabilized with 0.2% Triton X-100 (Sigma, USA) for 10 min at room temperature and then blocked with 0.3% bovine serum albumin (Abcam, UK) at 37°C for 1 h. After additional PBS washes, the cells were incubated with the primary antibodies—anti-HIF1α or BVDV NS5B—at 37°C for 1 h. The cells were washed again and incubated with a goat anti-mouse IgG-FITC antibody at 37°C for 1 h. Following three additional PBS washes, the fluorescence signals were visualized using a fluorescence microscope.

### RNA interference (RNAi)

Small-interfering RNAs (siRNAs) targeting the genes encoding HK2, VDAC1, IFNAR, LDHA, PDHA, and MCT1 were designed and synthesized by Suzhou GenePharma Co., Ltd. (China). For gene knockdown, MDBK cells at approximately 80% confluency in 6-well plates were transfected with the respective siRNAs (siHK2, siVDAC1, siIFNAR, siLDHA, siPDHA, and siMCT1) using Lipofectamine 3000 transfection reagent (L3000015; Invitrogen, USA) according to the manufacturer’s instructions, and then incubated at 37°C under 5% CO2 for 24 h. After transfection, the cells were infected with BVDV (MOI = 5) for 48 h. Cell samples were then collected for further analysis. A non-targeting siRNA was used as a negative control. RNAi experiments were conducted in three technical replicates.

#### Enzyme-linked immunosorbent assay

To quantify type I IFN production, culture supernatants were harvested from MDBK cells subjected to the indicated treatments. Levels of bovine IFN-β were measured using a Bovine IFN-β ELISA Kit (MM-3694801; Meimian, China) according to the manufacturer’s instructions.

### ROS measurement

MDBK cells were cultured in 12-well plates until reaching approximately 80% confluency and then infected with BVDV (MOI = 5) for 0, 12, 24, and 48 h. After removal of the culture medium, the cells were incubated with 10 µM DCFH-DA (TargetMol, USA) at 37°C for 30 min. Subsequently, the cells were washed three times with DMEM and examined using a fluorescence microscope.

### Inhibitor treatment

MDBK cells were cultured in 6-well plates until reaching ca. 80% confluency and then treated with the following inhibitors: 10 μmol/mL PX-478 (a HIF-1α inhibitor; TargetMol, USA), 1 mmol/L 4-phenylbutyric acid (4-PBA, an endoplasmic reticulum stress inhibitor; TargetMol, USA), 10 μmol/mL Fasentin (a GLUT-1 inhibitor; Abmole, USA), 10 μmol/mL acetylcysteine (NAC, a specific inhibitor of reactive oxygen species; TargetMol, USA), 10 μmol/mL UK5099 (a mitochondrial pyruvate carrier inhibitor; MEC, USA), sodium oxamate (an LDHA inhibitor; APExBIO, USA) at final concentrations of 10 and 20 mmol/L and 10 mmol/mL sodium dichloroacetate (DCA; a pyruvate dehydrogenase kinase inhibitor; MEC, USA). After inhibitor treatment, the cells were infected with BVDV (MOI = 5) for 48 h and then harvested for subsequent analysis. All experiments included three independent biological replicates, each with three technical replicates.

### Co-immunoprecipitation (Co-IP)

Co-IP assays were performed to examine the interactions between HK2, MAVS, and VDAC1 proteins. Briefly, BT cells at approximately 80% confluency in six-well plates were co-transfected with plasmids expressing HK2/VDAC1, MAVS, and RIG-I. After 24 h of culture at 37°C in 5% CO2 incubator, the cells were further transfected with poly(I:C) (Sigma, USA) for another 24 h. The cells were then harvested and lysed using ice-cold Cell Lysis Buffer (#9803, Cell Signaling Technology, USA), followed by centrifugation at 12,000 rpm for 15 min at 4°C. The supernatant was incubated with an anti-Flag IgG antibody overnight at 4°C with gentle agitation. Then, 30 μL of pre-washed Dynabeads protein G magnetic beads (10004D; Invitrogen, USA) were added and incubated for 2 h at 4°C. After three washes to remove non-specifically bound proteins, the immunoprecipitates were analyzed by western blot. In parallel, BT cells were either transfected with siHK2 or siVDAC1 for 24 h, followed by poly(I:C) transfection for another 24 h, or treated with SO or siLDHA, followed by exogenous lactate supplementation, prior to BVDV infection (MOI = 5) for 48 h. Cells were then lysed, and the supernatant was subjected to Co-IP using an anti-MAVS antibody, followed by western blot analysis with antibodies against HK2, VDAC1, or RIG-I. All experiments were performed in triplicate to ensure technical reproducibility.

### Biotin-labeling lactate pull-down assay

A biotinylated lactate pull-down assay was performed to examine the interaction between lactate and the MAVS protein. Briefly, BT cells were transfected with pCMV^3×Flag^-MAVS for 24 h, harvested, and the total protein was extracted and quantified using the BCA method. Subsequently, 5 μg of biotinylated lactate was incubated with 500 μg of total protein on ice for 10 min. Magnetic beads were then added to the mixture and incubated at 4°C for 2 h with gentle rotation. The beads were collected using a magnetic rack, washed three times, and the bound proteins were eluted and analyzed by western blot. All experiments were performed in three technical replicates.

### Cell viability analysis

The effect of UK5099, sodium oxamate, DCA, lactate, transfection reagent, and DMSO at their respective working concentrations on cell viability was evaluated utilizing Cell Counting Kit-8 (K1018; APExBIO, USA) according to the manufacturer’s instructions. All experiments were performed with three independent biological replicates, each consisting of three technical replicates.

### Statistical analysis

Data are presented as mean ± standard deviation (SD). Student’s *t*-test was used for statistical analysis. Tukey’s multiple-comparison tests and one-way analysis of variance (ANOVA) were conducted on the experimental data using GraphPad Prism V8.0. Significant differences are indicated with asterisks (*, *P* < 0.05; **, *P* < 0.01; ***, *P* < 0.001; ****, *P* < 0.0001).

## Data Availability

All relevant data are within the article and are available from the lead contact (Yigang Xu, yigangxu_china@sohu.com) upon reasonable request.

## SUPPLEMENTAL MATERIAL

Table S1 jvi.00320-26-s0001.docx Primers used for the qRT-PCR assay in this study.

ASM does not own the copyrights to Supplemental Material that may be linked to, or accessed through, an article. The authors have granted ASM a non-exclusive, world-wide license to publish the Supplemental Material files. Please contact the corresponding author directly for reuse.
