# Supplementary material for: Pestivirus bovine viral diarrhea virus infection induces ROS–HIF-1a axis-driven glycolytic reprogramming, which increases viral replication by impairing RIG-I-dependent type I interferon response
Source: J Virol. 2026 May 7;100(6):e00320-26. doi: 10.1128/jvi.00320-26 (PMC13288779; doi:10.1128/jvi.00320-26)
Supplement: Table S1 — Primers used for the qRT-PCR assay in this study. [file jvi.00320-26-s0001.docx]

**Table S1** Primers used for qRT-PCR assay in this study.

| Genes | Primer sequence (5’-3’) | Accession number |
| --- | --- | --- |
| *IFN-β* | F: GGTAGCCCTGTGCCTGATTTCATC | NM_174350.1 |
|  | R: AAGGCTCTGACGTTGTTGGAATCG |  |
| *IFNAR* | F: TTCCTGACTTGAAACCGCTGACTG | NM_174552.2 |
|  | R: CTGCTCCCTTTATTCCGCCTGTC |  |
| *HK2* | F: TGTGGTTGCTGTGGTGAACGATAC | XM_015473383.2 |
|  | R: TGCTGCCTGTGCCAACAATGAG |  |
| *VDAC1* | F: GACAGGGTACAAGCGGGAACATATC | NM_174485.4 |
|  | R: CCAGCCAGCCAACCTTCATAACC |  |
| *GLUT1* | F: ATCCTCATCGCCCAGGTGTT | NM_174602.2 |
|  | R: GGTTCTCCTCGTTGCGGTTA |  |
| *PFKP* | F: AGCACGAGGAGTTCTGTGTC | NM_001193220.3 |
|  | R: GATGCGGTCACACGTATCGG |  |
| *HIF-1α* | F: CCTCTGATCTCACGAGGGGT | NM_174339.3 |
|  | R: TCGACGTTCAGAACTTATCTTTTTC |  |
| *RIG-I* | F: CGTGGCAGAACAAATCAGACAATGG | XM_024996055.1 |
|  | R: GGCGACCGAGGTAGCAATTAGAATC |  |
| *MAVS* | F: TGGCAGGCTGGTATCTAGGATGG | NM_001046620.2 |
|  | R: CAAGGAGTTACTGTGGCTGATGGC |  |
| *LDHA* | F: TTCAGCTCGCTTCCGTTATCTCATG | NM_174099.2 |
|  | R: ACACCAGCAACATTCACTCCACTC |  |
| *PDHA* | F: AGTGAGTTACCGTACCCGAGAAGAG | NM_001101046.2 |
|  | R: ATTGCTGTTCACCATCCTGTCCTTG |  |
| *MCT1* | F: CCATCGGCTTCTCTTATGCGTTCC | NM_001037319.1 |
|  | R: ACTGCTGATAGGACCTCCACCATAC |  |
| *ISG20* | F: GGCCCGAGGGTGACATCAC | [XM_019984047.2](https://www.ncbi.nlm.nih.gov/nucleotide/XM_019984047.2?report=genbank&log$=nucltop&blast_rank=2&RID=PKWW3N2K016) |
|  | R: CAGCGCCTTGAAGTCGTGCT |  |
| *ISG15* | F: TCGCCCAGAAGATCAATGTG | [XM_019976941.2](https://www.ncbi.nlm.nih.gov/nucleotide/XM_019976941.2?report=genbank&log$=nucltop&blast_rank=18&RID=PKWY9NTT014) |
|  | R: AGCACCTCCCTGCTGTCAAG |  |
| *IFITM1* | F: CTACCGCCAAGTGCCTGAA | [NM_001078142.2](https://www.ncbi.nlm.nih.gov/nucleotide/NM_001078142.2?report=genbank&log$=nucltop&blast_rank=19&RID=PKX0Y82M014) |
|  | R: AATGAGAAGAACGATCGATCCAA |  |
| *IFITM3* | F: GCTTAAGGAGGAGCACGAGG | [XM_005895122.3](https://www.ncbi.nlm.nih.gov/nucleotide/XM_005895122.3?report=genbank&log$=nuclalign&blast_rank=35&RID=PKXAKKKC016) |
|  | R: TGAACAGGGACCACACGATG |  |
| *MX1* | F: ACATGATCGTCAAGTGCCGT | [XM_015473623.3](https://www.ncbi.nlm.nih.gov/nucleotide/XM_015473623.3?report=genbank&log$=nucltop&blast_rank=17&RID=PKX5GMX2014) |
|  | R: AAGGTCCCTGAAATGTGCGT |  |
| *OAS1* | F: CTCACAGAGTTCGGGTGTCC | [XM_070386066.1](https://www.ncbi.nlm.nih.gov/nucleotide/XM_070386066.1?report=genbank&log$=nucltop&blast_rank=13&RID=PKX7SSHZ014) |
|  | R: TGCCGTTTCTGGACCTCAAA |  |
| *β-actin* | F: GCCAACCGTGAGAAGATGAC | LT575466.1 |
|  | R: AGGCATACAGGGACAGCACA |  |
| BVDV *5’UTR* | F: AAACAAGGAGGGTAGCAACAG | KC695814.1 |
|  | R: TCTGCAAGCACCCTATCA |  |
